# Supplementary material for: Optimising Access Surgery in Senior Haemodialysis Patients (OASIS): study protocol for a multicentre randomised controlled trial
Source: BMJ Open. 2022 Feb 3;12(2):e053108. doi: 10.1136/bmjopen-2021-053108 (PMC8814743; doi:10.1136/bmjopen-2021-053108)
Supplement: Supplementary data [file bmjopen-2021-053108supp001.pdf]

Supplementary data 1: The Dusseux score with predictor definitions. [17]

Score item	Score points	Definition given in Dusseux article
Gender		
Male	1	
Female	0	
Age		
70-75	0	
75-80	2	
80-85	5	
≥85	9	
Diabetes		
Yes	2	
No	0	
Ischemic heart disease		history of coronary vascular disease, myocardial infarction, coronary artery bypass surgery, angioplasty, or abnormal angiography
Yes	2	
No	0	
PVD stage III or IV		Leriche-Fontaine classification
No	0	
Stage I or II	0	
Stage III or IV	5	
Cerebrovascular disease		
Yes	1	
No	0	
Chronic heart failure		New York Heart Association stages I–IV
No	0	
Stage I or II	2	
Stage III or IV	4	
Dysrhythmia		defined as atrial fibrillation and in the case of specific treatment with one or more antiarrhythmics, or the presence of a pacemaker or a cardiac implantable defibrillator
Yes	2	
No	0	
Chronic Respiratory disease		Including chronic respiratory failure, defined as a steady-state PaO2 o60mmHg regardless of the level of capnia, a sleep apnea syndrome or requiring oxygen therapy or home ventilatory assistance, and chronic obstructive pulmonary disease, defined as a cough with permanent or recurrent sputum, especially in the morning, 3 months/years for 2 consecutive years
Yes	2	
No	0	
Active malignancy		
Yes	5	
No	0	
Severe behavioural disorder		including dementia, psychosis, or severe neurosis that may affect patient dependence or compliance with treatment
Yes	6	
No	0	
Mobility		
Walks without help	0	
Needs assistance for transfers	4	
Totally dependent for transfers	9	
BMI		
<21	3	
21-25	1	
≥25	0	
CVC at dialysis initiation		
Yes	3	
No	0	
