# Supplementary material for: Optimising Access Surgery in Senior Haemodialysis Patients (OASIS): study protocol for a multicentre randomised controlled trial
Source: BMJ Open. 2022 Feb 3;12(2):e053108. doi: 10.1136/bmjopen-2021-053108 (PMC8814743; doi:10.1136/bmjopen-2021-053108)
Supplement: Author's manuscript [file bmjopen-2021-053108.draft_revisions.pdf]

BMJ Open

BMJ Open is committed to open peer review. As part of this commitment we make the peer review history of every article we publish publicly available.

When an article is published we post the peer reviewers' comments and the authors' responses online. We also post the versions of the paper that were used during peer review. These are the versions that the peer review comments apply to.

The versions of the paper that follow are the versions that were submitted during the peer review process. They are not the versions of record or the final published versions. They should not be cited or distributed as the published version of this manuscript.

BMJ Open is an open access journal and the full, final, typeset and author-corrected version of record of the manuscript is available on our site with no access controls, subscription charges or pay-per-view fees (http://bmjopen.bmj.com).

If you have any questions on BMJ Open's open peer review process please email info.bmjopen@bmj.com

BMJ Open

Optimising Access Surgery In Senior haemodialysis patients (OASIS): Study protocol for a multicentre randomised controlled trial

Journal:	*BMJ Open*
Manuscript ID	bmjopen-2021-053108
Article Type:	Protocol
Date Submitted by the Author:	04-May-2021

Complete List of Authors:	Heggen, Boudewijn; Maastricht University Medical Centre+, Department of Vascular Surgery<br>Ramspek, Chava; Leiden University Medical Center, Department of Clinical Epidemiology<br>van der Bogt, Koen; Haaglanden Medical Centre, Department of Surgery; Leiden University Medical Center, Department of Surgery<br>de Haan, Michiel; Maastricht University Medical Center, Department of Radiology<br>Hemmelder, Marc; Maastricht University Medical Center, Department of Internal Medicine, division of nephrology<br>Hiligsmann , Mickaël ; Maastricht University, Department of Health Services Research, CAPHRI Care & Public Health Research Institute<br>van Loon, Magda; Maastricht University Medical Center, Department of Vascular Surgery<br>Rotmans, Joris; Leiden University Medical Center, Department of Internal Medicine<br>Tordoir, Jan; Maastricht University Medical Center, Department of Vascular Surgery<br>Dekker, Friedo; Leiden University Medical Centre, Department of Clinical Epidemiology<br>Schurink, Geert-Willem; Maastricht Universitair Medisch Centrum+, Department of Vascular Surgery<br>Snoeijs, Maarten; Maastricht University Medical Center, Department of Vascular Surgery

Keywords:	VASCULAR SURGERY, Vascular surgery < SURGERY, Dialysis < NEPHROLOGY, End stage renal failure < NEPHROLOGY, Adult nephrology < NEPHROLOGY

SCHOLARONE™
Manuscripts

Optimising Access Surgery In Senior haemodialysis patients (OASIS): Study protocol for a multicentre randomised controlled trial

Boudewijn DC Heggen[1], Chava L Ramspek[2], Koen EA van der Bogt[3,4], Michiel W de Haan[5], Marc H Hemmelder[6], Mickaël JC Hiligsmann[7], Magda M van Loon[1], Joris I Rotmans[8], Jan HM Tordoir[1], Friedo W Dekker[2], Geert Willem H Schurink[1], Maarten GJ Snoeijs[1], on behalf of the OASIS study group[9].

1 Department of Vascular Surgery, Maastricht University Medical Center, Maastricht, The Netherlands.
2 Department of Clinical Epidemiology, Leiden University Medical Center, Leiden, The Netherlands.
3 Department of Surgery, Haaglanden Medical Center, The Hague, The Netherlands.
4 Department of Surgery, Leiden University Medical Center, Leiden, The Netherlands.
5 Department of Radiology, Maastricht University Medical Center, Maastricht, Netherlands.
6 Department of Internal Medicine, division of nephrology, Maastricht University Medical Center, Maastricht, The Netherlands.
7 Department of Health Services Research, CAPHRI Care & Public Health Research Institute, Maastricht University, Maastricht, The Netherlands.
8 Department of Internal Medicine, Leiden University Medical Center, Leiden , The Netherlands.
9 Members of the OASIS study group are listed at the end of the article.

Corresponding author

Name: Boudewijn DC Heggen
Post address: P.0. box 5800, 6202 AZ, Maastricht, The Netherlands, Maastricht University Medical Center, Department of Vascular Surgery.
Email: boudewijn.heggen@mumc.nl

Word count (main text)
3998

For peer review only

ABSTRACT

Introduction

Current evidence on vascular access strategies for haemodialysis patients is based on observational studies that are at high risk of selection bias. For elderly patients, the autologous arteriovenous fistulas that are recommended by current guidelines may not be the best option because a significant proportion of fistulas either fail to mature or remain unused. In addition, long-term complications associated with arteriovenous grafts and central venous catheters may be less relevant when considering the limited life expectancy of these patients. Therefore, we designed the OASIS trial to determine the best strategy for vascular access creation in elderly haemodialysis patients.

Methods and analysis

OASIS is a multicentre randomised controlled trial with an equal participant allocation in three treatment arms. Patients aged 70 years or older who are expected to initiate haemodialysis treatment in the next 6 months or who have started haemodialysis urgently with a catheter will be enrolled. To detect and exclude patients with an unusually long life expectancy, a previously published mortality prediction model will be used after external validation. Participants allocated to the usual care arm will be treated according to current guidelines on vascular access creation and will undergo fistula creation. Participants allocated to one of the two intervention arms undergo graft placement or catheter insertion. The primary outcome is the number of access-related interventions required for each patient-year of haemodialysis treatment. We will enrol 195 patients to have sufficient statistical power to detect an absolute decrease of 0.80 interventions per year.

Ethics and dissemination

Because of clinical equipoise, we believe it is justified to randomly allocate elderly patients to the different vascular access strategies. The study was approved by an accredited Medical Ethics Review Committee. The results will be disseminated through peer-reviewed publications and will be implemented in clinical practice guidelines.

Trial registration

Netherlands trial register, NL7933, registered on August 4th, 2019.

Protocol version and date

Version 5, February 25th 2021

Key words

Vascular access, haemodialysis, elderly, arteriovenous fistula, central venous catheter, arteriovenous graft.

ARTICLE SUMMARY

Strengths and limitations of this study

- This is the first prospective trial randomly allocating elderly haemodialysis patients to the three commonly used vascular access strategies.
- By enrolling patients upon referral for vascular access creation, the trial will reflect everyday practice and study patients with pre-dialysis vascular access creation as well as patients who started dialysis urgently with a temporary catheter.
- The primary outcome, i.e. the number of access-related interventions required for each year of haemodialysis treatment, corresponds to the recently proposed core outcome measure for clinical trials in vascular access.
- A previously published mortality prediction score was validated to determine a cut-off point for exclusion of elderly patients with an unusually long life expectancy for whom a fistula is still considered to be the best vascular access.

For peer review only

INTRODUCTION

The haemodialysis population has been growing older and older over the past decades, and the median age of patients starting haemodialysis treatment today is 66 years.[1] These patients need a reliable vascular access that may be provided by autologous arteriovenous fistulas, prosthetic arteriovenous grafts, and central venous catheters. Autologous fistulas are created by surgically anastomosing a superficial vein to an artery in the upper extremity. After creation of the fistula, vascular remodelling will increase venous outflow diameter and blood flow, a process referred to as maturation. Functional fistulas are generally associated with the best long-term outcomes. However, a substantial proportion of fistulas fail to mature and never become functional.[2] Arteriovenous grafts are placed by interposing a prosthetic vascular graft between an artery and a vein in a subcutaneous track in the upper extremity. Grafts do not need time to mature, but are associated with a higher incidence of infection, stenosis, and thrombosis than functional fistulas.[3] Central venous catheters are inserted in the internal jugular vein with a minimally invasive procedure and can be used immediately after placement. However, catheters have been associated with increased bloodstream infections and mortality compared to other vascular access types.[3, 4] Studies comparing these different vascular access strategies typically focus on functional outcomes, and little is known about differences in patient reported outcome measures and health care costs.

Clinical practice guidelines on vascular access have long recommended the creation of autologous arteriovenous fistulas regardless of patient characteristics.[5] However, this recommendation is based solely on observational studies that are at high risk of bias. Patients who have a fistula created prior to starting dialysis generally have more favourable baseline characteristics and a lower mortality risk than other patients.[6] These favourable patient characteristics may account for two-thirds of the survival benefit seen in patients using arteriovenous fistulas for haemodialysis treatment instead of central venous catheters.[7] Further selection bias will occur as patients are analysed according to vascular access use. This tends to favour autologous fistulas since the poor subset of patients with fistulas that have failed to mature will go on to use a different type of vascular access. When primary fistula failures are taken into account, the cumulative patency of fistulas and grafts become comparable.[8] Finally, observational studies based on dialysis registries do not include patients with arteriovenous fistulas created before dialysis initiation that were never used because of patient death or stabile renal function.

Uncertainty regarding the best type of vascular access is particularly important for elderly patients starting haemodialysis treatment. In these patients, autologous arteriovenous fistulas have a 40% increased risk of non-maturation and early failure.[2] Moreover, 30% of fistulas created before initiation of dialysis treatment in elderly patients will never be used because of patient death, stabilization of renal function, or choice for conservative treatment as the patient's condition worsens over time.[9] Furthermore, the increase in long-term complications associated with arteriovenous grafts may be less relevant for elderly patients with a limited life expectancy. Finally, elderly patients have a 30% lower risk of complications of central venous catheters, including bloodstream infections.[4] Considering this clinical equipoise, the need for better evidence on vascular access strategies in elderly haemodialysis patients is broadly recognised. Indeed, international experts on vascular access surgery as well as patient representatives have called for a randomised trial comparing vascular access strategies in elderly haemodialysis patients.[5, 10]

We performed a comprehensive search in international trial registers for ongoing and planned randomised trials on vascular access creation in elderly haemodialysis patients. Four ongoing trials were identified that allocate participants to arteriovenous fistula or graft surgery. One trial has a single centre design and plans to enrol 270 haemodialysis patients aged 70 years or older. The participants are followed for two years after vascular access creation for the primary outcomes, which are vascular access use as well as patency.[11] The remaining three trials are pilot studies evaluating the feasibility of the trial design. These pilot studies allocate patients aged 65 years or older, either at the time of

referral for vascular access, at dialysis initiation, or after dialysis initiation with a catheter.[12-14] We also identified two trials allocating participants to fistula creation or catheter insertion. The ACCESS HD feasibility study allocates patients aged 55 years or older who have already commenced haemodialysis treatment with a catheter to either fistula creation or continued catheter use.[10] The AUSTRIA study aims to allocate 220 participants aged 60 years or older to one of the two treatment arms, with access-related complications being the primary outcome measure.[15] Although these ongoing studies will surely provide valuable information, none of the trials cover the full spectrum of vascular access strategies in elderly haemodialysis patients.

The aim of the OASIS trial is to determine the best vascular access strategy for elderly haemodialysis patients. We hypothesize that arteriovenous grafts and central venous catheters are superior to autologous arteriovenous fistula for elderly patients starting haemodialysis treatment and result in reduced access-related intervention rates, greater patient satisfaction, and lower health care costs.

METHODS AND ANALYSIS

Study design

OASIS is a parallel group, multicentre, randomised controlled trial, with a superiority framework and a 1:1:1 individual participant treatment allocation ratio over 3 study arms. Given the nature of the interventions, blinding health care providers, participants, or outcome assessors for treatment assignment is not possible. However, data analysis will be performed without knowledge of treatment assignment by coding the treatment arms and unlocking the code only after statistical analysis is completed. Some 17 hospitals, together treating approximately 50% of all haemodialysis patients in the Netherlands, are expected to participate.

Study population

Upon referral for permanent vascular access creation, all patients will have a preoperative duplex ultrasound assessment of the blood vessels of the upper extremities. This preoperative workup may be supplemented by central venous phlebography and arterial imaging as considered appropriate by the local vascular access team. All potential participants will receive oral and written information about the trial by the local investigators and are registered in a screening log.

Inclusion criteria:

1. Age 70 years or older;
2. End-stage renal disease with unlikely recovery of kidney function according to the attending nephrologist;
3. Haemodialysis is the intended long-term modality of treatment for end-stage renal disease;
4. Fit for vascular access surgery as determined by the local multidisciplinary vascular access team;
5. a. Expected to start haemodialysis treatment within 6 months at the time of treatment assignment; or
   b. Treated with haemodialysis for 6 months or less at the time of treatment assignment using a tunnelled or non-tunnelled central venous catheter for vascular access;
6. Planning to remain in one of participating dialysis centres for at least 1 year;
7. Suitable vascular anatomy for all types of vascular access based on duplex ultrasound of the arms, defined as:
   - at least one suitable configuration for an arteriovenous fistula using minimal arterial and venous diameters of 2mm for radiocephalic fistulas and 3mm for brachiocephalic and brachiobasilic fistulas;
   - at least one suitable configuration for an arteriovenous graft using minimal arterial and venous diameters of 3mm and 4mm, respectively; and
   - at least one open internal jugular vein for a central venous catheter.

For peer review only - http://bmjopen.bmj.com/site/about/guidelines.xhtml

Exclusion criteria:

1. Patent arteriovenous fistula or graft already in place;
2. Prior unsuccessful arteriovenous fistula or graft vascular access surgery;
3. Kidney transplantation planned within 6 months;
4. Metastatic malignancies or other condition associated with a life expectancy of <6 months, in the opinion of the attending nephrologist;
5. Unable to provide informed consent;
6. Dusseux risk score ≤ 4, indicating an unusually long life expectancy.[16]

Patients will be enrolled by local investigators if they meet the eligibility criteria and provide informed consent. Local investigators will be informed of treatment assignment through a web-based service, after which vascular access creation is planned according to usual clinical care for the allocated vascular access type at the study site (Figure 1). The treatment allocation sequence is generated by an independent data management centre using a computer-based random number producing algorithm. Block randomisation is implemented with randomly varying block sizes of 3 and 6, and randomisation will be stratified by treatment centre.

External validation of a mortality prediction model

Elderly haemodialysis patients form a heterogeneous population in terms of comorbidities and life expectancy. The majority of elderly haemodialysis patients have multiple comorbidities and a relatively short life expectancy making the long-term performance of the vascular access less relevant. However, for the small group of elderly patients with an unusually long life expectancy, autologous arteriovenous fistulas are considered to be the best vascular access given their low complication rates after maturation. To identify this subset of elderly patients that should be excluded from randomisation, we performed an external validation of the mortality prediction score developed by Dusseux et al.[16]. This prediction score contains items readily identified from the medical history and can be completed before initiation of haemodialysis treatment.

The mortality prediction score was validated in the Netherlands Cooperative Study on the Adequacy of Dialysis (NECOSAD).[17, 18] In this multicentre observational cohort study, adult patients starting dialysis treatment between 1997 and 2007 in the Netherlands were prospectively followed until February 1st, 2015. For the current validation, we restricted the sample to haemodialysis patients aged 70 years and older. This resulted in 494 patients, 411 of whom died during follow-up with a median survival of 2.3 years (interquartile range: 1.1-4.4 years). Overall, baseline characteristics in the validation cohort were comparable to those in the Dusseux cohort (table 1).[16] Missing data were assumed to be largely missing at random. Therefore, 10-fold multiple imputation with fully conditional specification was performed using the R package 'mice'. All variables of the mortality prediction score and the outcome were included in the imputation model.[19, 20]

Each individual's risk score was calculated with the original point system as presented by Dusseux (supplementary data 1). Risk scores ranged from 0 to 28, with a higher score corresponding to a higher risk of mortality. From the individual risk scores, the discrimination for mortality within 2 years was assessed by c-statistics that were pooled over the ten imputation datasets according to Rubin's Rules.[21] The Dusseux mortality risk score had a c-statistic of 0.68 (95% CI: 0.63-0.73) in the NECOSAD validation cohort. Furthermore, the cohort was divided into 5 groups with increasing mortality risk. A calibration plot of predicted versus observed mortality risk was computed. Calibration was moderate as the model tended to overpredict the mortality risk (53% average predicted risk and 38% average observed risk; Figure 2). Finally, we determined a sensible cut-off point for identifying a subset of patients with an unusually long life expectancy. A cut-off between a score of ≤4 and ≥5 was chosen to separate patients with unusually good outcomes (consisting of 22% of patients in the validation cohort with a median survival of 5.0 years (95% CI: 4.3-5.4)) from elderly haemodialysis patients with a poor

For peer review only

life expectancy (consisting of the remaining 78% of patients in the validation cohort with a median survival of 2.2 years (95% CI: 1.9-2.5); Figure 3).

	**NECOSAD cohort n=494**	**Dusseux cohort n=8955**
Gender (male)	61 %	60 %
Age	76 (73 – 79)	78 (74 – 82)
Diabetes	25 %	38 %
Ischemic heart disease[1]	31 %	14 %
Peripheral vascular disease	24 %	27%
Cerebrovascular disease	14 %	12 %
Congestive heart failure	19 %	34 %
Dysrhythmia[2]	22 %	25 %
Respiratory disease[3]	12%	13 %
Active malignancy	4 %	11 %
Psychiatric disorder[4]	3 %	4 %
Mobility[5, 6]		
- Walks without help	64 %	69 %
- Needs assistance for transfers	32 %	22 %
- Totally dependent for transfers	4 %	9 %
BMI (Median)[7]		
- < 21 kg/m^2	13 %	18 %
- 22 – 25 kg kg/m^2	43 %	35 %
- 25 – 30 kg/m^2	33 %	33 %
- > 30 kg/m^2	10 %	14 %
CVC at dialysis initiation	23 %	42 %

Table 1: Baseline characteristics of the NECOSAD validation cohort compared to the Dusseux derivation cohort.[16] 1: Ischemic heart disease was defined as a history of angina pectoris or myocardial infarction. 2: Vitamin K antagonist use was used as a proxy for cardiac dysrhythmia. 3: Respiratory disease was defined as the need to take pulmonary medication on a daily basis. 4: Psychiatric disorder was defined as dementia, depression, or other psychiatric disease. 5: The Karnofsky score was used as a proxy for mobility, where a score of >60 was considered as 'walks without help', a score of 50-60 as 'needs assistance for transfers', and a score <50 as 'totally dependent for transfers'. 6: 32% of the data concerning mobility was missing in the Dusseux cohort. 7: 29% of the data concerning BMI was missing in the Dusseux cohort.

Interventions

The surgical strategies in the usual care and intervention groups are part of standard clinical care. Because we aim to compare the different surgical strategies for vascular access creation in everyday practice, we will not interfere with surgical protocols at the study sites. However, we expect the study sites to follow current best practice and guideline recommendations.[5]

Autologous arteriovenous fistulas

Fistulas are preferably created at the most distal site with adequate blood vessels, in the non-dominant arm. It is recommended to create the fistula 3 to 6 months before the expected start of haemodialysis treatment. In case of fistula failure, a new arteriovenous fistula may be created at the next available site.

For peer review only

Prosthetic arteriovenous grafts

Patients who are allocated to the graft strategy will have a commercially available prosthetic vascular graft implanted for haemodialysis access. It is recommended to place the arteriovenous graft two weeks before the expected start of haemodialysis treatment with antibiotic prophylaxis. Placement of an early-cannulation graft is recommended for patients who require more urgent start of haemodialysis to avoid the use of a temporary central venous catheter. In case of graft failure despite thrombectomy, a new graft may be placed.

Central venous catheters

Patients who are allocated to the catheter strategy will have a dialysis catheter inserted, unless they have already started haemodialysis with a tunnelled catheter. We recommend placing a tunnelled catheter just before the start of haemodialysis treatment. We advise to follow published guidelines for the prevention and treatment of catheter infections.[22] In case of catheter dysfunction despite local thrombolytic agents, the catheter can be exchanged.[23]

For the arteriovenous fistula and graft group, surveillance and pre-emptive intervention for stenosis will take place according to usual practice at the study sites. In case of failure, both endovascular and surgical interventions can be undertaken to restore patency. If the assigned vascular access strategy proves not to be feasible, patients will crossover to one of the other treatment groups after discussion of the case with the principal investigator. Use of a temporary central venous catheter is not considered as a treatment group crossover.

Primary study outcome

The primary study outcome is the number of access-related interventions required for each person-year of haemodialysis treatment. The primary outcome corresponds to the proposed core outcome measure for haemodialysis vascular access.[24] This outcome measure includes all percutaneous access interventions, surgical access procedures, and catheter interventions from randomisation until the end of the study period or death. The outcome measure specifically includes interventions before dialysis initiation and after dialysis cessation in the occasional patient who stops haemodialysis treatment after kidney transplantation, peritoneal dialysis, recovery of renal function, or refusal of further haemodialysis. Patients who discontinue haemodialysis treatment will therefore remain in the trial. Access-related complications that are resolved using conservative or pharmacological treatment are not counted as interventions.

Secondary study outcomes

Complications

Access-related complications requiring pharmacological, surgical, or endovascular interventions will be registered from randomisation until the end of the study period or death. Furthermore, the number of days admitted to hospital or visiting out-patient clinics for any reason and for vascular access-related reasons per person-year will be registered. Finally, all-cause mortality from randomisation to the end of the study period will be registered.

Patient-reported outcome measures (PROMs)

1. Short-Form Health Survey (SF-12) and the Dialysis Symptom Index (DSI) measure generic health-related quality of life as well as disease-specific symptom burden.[25]
2. The Short-Form Vascular Access Questionnaire (SF-VAQ) measures haemodialysis patients' satisfaction with their vascular access.[26]
3. The 5-level EuroQol 5-dimensional questionnaire (EQ-5D-5L) measures health-state utility values.[27]

The SF-VAQ will be administered on a monthly basis, whereas the SF-12, DSI and EQ-5D-5L will be administered at 3-month intervals. The questionnaires will be administered from randomisation until the one year after study or dialysis initiation, whichever is later.

For peer review only

Other outcome measures will be registered for exploratory analyses and are defined according to the ESVS guidelines on vascular access:[5]

1. Primary, assisted primary, secondary, and functional patency of vascular access.
2. Time until vascular access maturation (definition according to American Society of Nephrology).[28]
3. Time until functional vascular access.
4. The number of haemodialysis sessions with inadequate haemodialysis dose (Kt/V) and with cannulation difficulties or failure.

Quality and safety

Data is entered on electronic case report forms by trained investigators using numerical codes that cannot be traced to the individual subjects. The subject identification code list is kept by the principle investigator at each study site. Any serious adverse events that may occur will be reported to the principle investigator without undue delay after obtaining knowledge of the events. To evaluate differences in serious adverse events and mortality between treatment arms, an interim safety analysis will be performed and presented to a Data Safety Monitoring Board every six months. Significant differences on safety outcomes may lead to discontinuation of the trial. Furthermore, an independent monitor will be appointed to assess trial processes and data.

Sample size calculation

Sample size was estimated for the primary study outcome of access-related intervention rates. Since two intervention groups will be compared to the usual care group, we used Bonferroni correction to account for multiple comparisons, which resulted in an alpha of 0.025. The average number of interventions per year for patients receiving an autologous arteriovenous fistula has previously been reported at 2.48 interventions per year.[29] The clinically relevant effect size was considered to be 1 percutaneous intervention per year and 0.5 surgical interventions per year. Since these interventions occur at a ratio of 1:0.7,[29] a reduction of the overall intervention rate by 0.80 interventions per year was used as the clinically relevant effect for sample size calculations.

With these assumptions, a total number of 195 patients (65 patients in each treatment arm) with one year of follow-up achieves 80% power to detect a 0.80 decrease in the number of access-related interventions per person-year of haemodialysis treatment between the intervention groups and the usual care group using a two-sided, large-samples z-test of the Poisson event-rate difference at a significance level of 0.025.[30]

Since the study includes patients with a limited life expectancy, a substantial proportion of participants is expected to die before contributing one year of follow-up. To compensate for the resulting loss of statistical power, the trial has a variable follow-up with trial closeout for all patients at the time when the last patient enrolled has one year of follow-up. Since participant recruitment is expected to take 2 years, the resulting additional follow-up time will more than compensate for the loss of follow-up due to patient mortality in the first year of the study period.

Data analysis plan

The access-related intervention rates will be analysed using a general linear model with Poisson distribution and identity link, and with time as off-set variable. Both intervention groups will be compared to the usual care group. Non-adherence to the allocated treatment group is expected as patients cross over to another surgical strategy for vascular access when the randomised strategy is no longer feasible. The primary analysis will be on the intention to treat population. Exploratory on-treatment analyses will be performed as well. A subgroup analysis will be performed for patients who had already started haemodialysis using a catheter before inclusion.

For peer review only

Formal statistical comparisons will be made for the following secondary study outcomes:
1. SF-12 / DSI and SF-VAQ summary scores using generalised estimating equations.
2. The number of access-related serious adverse events per person-year using general linear models with Poisson distribution, and mortality using Cox regression.
3. The number of days admitted to hospital or visiting out-patient clinics for any reason per person-year using student t-tests.

Primary outcome data are not expected to be missing, as interventions on vascular access will be reported in the patients' medical files. We expect little to no loss to follow-up since study participants will be observed three times per week in the dialysis unit and the trial requires no additional study visits. Patient-reported outcome measures will be analysed using generalised estimating equations that allow for missing data. Other missing data will be handled by using 10 imputation cycles with regression methods, performing standard analyses for each imputation cycle, and considering the variability across the imputation cycles in the final analysis.

Economic analysis
A cost effectiveness analysis and a budget impact analysis comparing the different treatment strategies will be performed by collecting financial data from randomisation until the one year after study or dialysis initiation, whichever is later. The economic analyses will be done according to Dutch national guidelines and have a time horizon of 2 years that corresponds to the median life expectancy of the trial participants.[31]

For the cost effectiveness analysis, we will consider both healthcare sector costs and costs for patients and their families. Due to the age of our study population, absenteeism in paid work and production losses in the domestic sphere will not be considered. The relevant cost factors for the healthcare sector will be derived from hospital and pharmacy registration systems at the individual participant level. Costs to patients and families will be measured using a study-specific adaptation of the Medical Consumption Questionnaire developed by the Institute for Medical Technology Assessment at 3-month intervals.[32] After measurement of the relevant cost factors, monetary values will be assigned.

The budget impact analysis will be performed to estimate the financial consequences for the Dutch national health care budget for the different vascular access strategies in elderly haemodialysis patients. The perspectives that will be included in the analysis are the wider societal perspective including productivity losses, the narrower perspective of the public, and the perspective of health care providers and health insurance companies. Several scenarios will be included to assess the impact of different reimbursement options.

Patient and public involvement
The Dutch Renal Patients Society (NVN) was involved in the design of OASIS. We consulted patient representatives on the feasibility of randomisation into the three treatment arms and on the choice of the primary outcome measure. Patient representatives also advised on the frequency and duration of patient-reported outcome measurements and approved the patient information letter.

ETHICS AND DISSEMINATION
Ethics
The study will be conducted according to the principles of the Declaration of Helsinki (Brazil, 2013) and the Medical Research Involving Human Subjects Act (WMO). The protocol was approved by an accredited Medical Ethics Review Committee in November 2019. Some physicians may consider it unethical to use central venous catheters in haemodialysis patients who are candidates for autologous arteriovenous fistula creation, considering the increased mortality and infection rate associated with catheter use. However, there is serious doubt whether a causal relationship between catheter use and mortality exists. The association between catheter use and mortality is derived from observational

For peer review only

studies that are profoundly influenced by selection bias. Two examples of this are found in recent studies demonstrating that patient selection for fistula placement, even when they are dialysed with a catheter instead, explains at least two thirds of the mortality benefit observed in patients with a fistula.[6, 7] Furthermore, the majority of the excess mortality observed in patients who use a catheter is felt to be attributable to catheter-related bloodstream infections. However, during the run-in period of a multicentre randomised controlled trial, the incidence of catheter-related bloodstream infections was only 1.0 per 1000 catheter days.[33] In elderly patients, the risk of infection is even lower than in the general haemodialysis population.[9] In a large cohort of dialysis patients with catheter-related bloodstream infections, only 1% of these infections were fatal.[34] Therefore, the observed survival benefit with fistulas can impossibly be explained by avoidance of access-related infections. Moreover, there is clinical equipoise in elderly haemodialysis patients because the balance between risks and benefits of the different types of vascular access is different in this population. Any increase in access-related complications associated with long-term use of grafts and catheters may be less relevant for elderly patients. Finally, our study has been designed to minimise the potential risk for participants by scheduling frequent safety analyses, by allowing treatment crossover in case of access complications, and by excluding elderly patients with an unusually long life expectancy.

Dissemination

The OASIS trial is expected to lead to new insights in vascular access surgery for elderly haemodialysis patients. After completion of the trial, the results will be disseminated among vascular surgeons, nephrologists, interventional radiologists, and dialysis nurses through international peer-reviewed publications in medical journals and presentations at scientific meetings.

After the trial results have been published, its findings should be implemented into clinical practice. To identify possible facilitators and obstacles for implementation, OASIS will include a process evaluation. We will register the reasons for patients not participating in the randomised trial in a screening log and we will study ineligible elderly haemodialysis patients in a parallel observational cohort study. Patient characteristics and outcomes of the randomised trial and the observational cohort will be compared to evaluate the generalisability of the trial. Furthermore, we will register study protocol violations during the enrolment and follow-up phase and we will investigate any protocol violations, such as non-adherence to the assigned treatment, by interviewing the treating physician. This process evaluation will refine our implementation plan with strategies tailored to the specific clinical circumstances.

Author contributions

BDCH drafted the manuscript. MGJS designed the study and supervised the writing of the manuscript. CLR and FWD performed the external validation of the mortality prediction score. KEAB, MWH, MHH, MJCH, MML, JIR, JHMT and GWHS provided significant intellectual input in the development of the study protocol. All authors read and approved the final manuscript.

Competing interests

None declared.

Funding statement

This study was funded by Leading the Change (ZonMw project number 80-85009-98-2004). The funder has no role in the design and management of the trial, nor in the data collection and analysis or interpretation and publication of the results.

Data statement

After completion of the trial, data will be made available for further research and verification and will therefore be documented and saved in an accessible manner that is in accordance with the laws and regulations concerning privacy sensitive data.

Group information

Members of the OASIS study group: Marijke J Molegraaf (Department of Vascular Surgery, Isala, Zwolle, The Netherlands); Philippe WM Cuypers (Department of Vascular Surgery, Catharina Ziekenhuis, Eindhoven, The Netherlands). Denise Nio (Department of Vascular Surgery, Spaarne Gasthuis, Haarlem, The Netherlands); Cora H Arts (Department of Vascular Surgery, Medisch Centrum Leeuwarden, Leeuwarden, The Netherlands); Jacobien C Verhave (Department of Internal Medicine, Rijnstate, Arnhem, The Netherlands); Çagdas Ünlü (Department of Vascular Surgery, Noordwest Ziekenhuisgroep, Alkmaar, The Netherlands); Patrick WHE Vriens (Department of Vascular Surgery, Elisabeth-TweeSteden Ziekenhuis, Tilburg, The Netherlands); Goos D Laverman (Department of Internal Medicine, Ziekenhuisgroep Twente, Almelo, The Netherlands); Aron S Bode (Department of Vascular Surgery, Canisius Wilhelmina Ziekenhuis, Nijmegen, The Netherlands); Marcel C Weijmer (Department of Internal Medicine, OLVG, Amsterdam, The Netherlands); Bas Govaert (Department of Vascular Surgery, Máxima Medisch Centrum, Veldhoven, The Netherlands); Aaltje Y Adema (Department of Internal Medicine, Medisch Centrum Leeuwarden, Leeuwarden, The Netherlands); Gijs MJM Welten (Department of Vascular Surgery, Franciscus Gasthuis & Vlietland, Rotterdam, the Netherlands); Michel MPJ Reijnen (Department of Vascular Surgery, Rijnstate, Arnhem, The Netherlands); Marc JH Groeneveld (Department of Internal Medicine, Haaglanden Medisch Centrum, The Hague, The Netherlands); Hans S Brink (Department of Internal Medicine, Medisch Spectrum Twente, Enschede, The Netherlands); Jeroen B van der Net (Department of Internal Medicine, Albert Schweitzer Ziekenhuis, Dordrecht, The Netherlands); Arno Kuijper (Department of Internal Medicine, Máxima Medisch Centrum, Veldhoven, The Netherlands); Hilde PE Peters (Department of Internal Medicine, Isala, Zwolle, The Netherlands); Maarten A Lijkwan (Department of Vascular Surgery, Albert Schweitzer Ziekenhuis, Dordrecht, The Netherlands); Edith M Willigendael (Department of Vascular Surgery, Medisch Spectrum Twente, Enschede, The Netherlands); Roos C van Nieuwenhuizen (Department of Vascular Surgery, OLVG, Amsterdam, The Netherlands); Rombout R Kruse (Department of Vascular Surgery, Ziekenhuisgroep Twente, Almelo, The Netherlands);); Yvonne C Schrama (Department of Internal Medicine, Franciscus Gasthuis & Vlietland, Rotterdam, the Netherlands); Karina A de Groot (Department of Internal Medicine, Noordwest Ziekenhuisgroep, Alkmaar, The Netherlands); Karima Farhat (Department of Internal Medicine, Spaarne Gasthuis, Haarlem/Hoofddorp, The Netherlands).

Supplementary materials

- Supplementary data 1: Point system as presented by Dusseux[16]

References

1. Kramer A, Boenink R, Noordzij M, et al. The ERA-EDTA Registry Annual Report 2017: a summary. *Clin Kidney J* 2020;13(4):693-709. doi: 10.1093/ckj/sfaa048
2. Bylsma LC, Gage SM, Reichert H, et al. Arteriovenous Fistulae for Haemodialysis: A Systematic Review and Meta-analysis of Efficacy and Safety Outcomes. *Eur J Vasc Endovasc Surg* 2017;54(4):513-22. doi: 10.1016/j.ejvs.2017.06.024
3. Almasri J, Alsawas M, Mainou M, et al. Outcomes of vascular access for hemodialysis: A systematic review and meta-analysis. *J Vasc Surg* 2016;64(1):236-43. doi: 10.1016/j.jvs.2016.01.053
4. Poinen K, Quinn RR, Clarke A, et al. Complications From Tunneled Hemodialysis Catheters: A Canadian Observational Cohort Study. *Am J Kidney Dis* 2019;73(4):467-75. doi: 10.1053/j.ajkd.2018.10.014

For peer review only

5. Schmidli J, Widmer MK, Basile C, et al. Editor's Choice - Vascular Access: 2018 Clinical Practice Guidelines of the European Society for Vascular Surgery (ESVS). *Eur J Vasc Endovasc Surg* 2018;55(6):757-818. doi: 10.1016/j.ejvs.2018.02.001
6. Quinn RR, Oliver MJ, Devoe D, et al. The Effect of Predialysis Fistula Attempt on Risk of All-Cause and Access-Related Death. *J Am Soc Nephrol* 2017;28(2):613-20. doi: 10.1681/asn.2016020151
7. Brown RS, Patibandla BK, Goldfarb-Rumyantzev AS. The Survival Benefit of "Fistula First, Catheter Last" in Hemodialysis Is Primarily Due to Patient Factors. *J Am Soc Nephrol* 2017;28(2):645-52. doi: 10.1681/asn.2016010019
8. Lok CE, Sontrop JM, Tomlinson G, et al. Cumulative patency of contemporary fistulas versus grafts (2000-2010). *Clin J Am Soc Nephrol* 2013;8(5):810-8. doi: 10.2215/cjn.00730112
9. Lee T, Thamer M, Zhang Y, et al. Outcomes of Elderly Patients after Predialysis Vascular Access Creation. *J Am Soc Nephrol* 2015;26(12):3133-40. doi: 10.1681/ASN.2014090938
10. Quinn R, Ravani P. ACCESS HD pilot: A randomised feasibility trial Comparing Catheters with fistulas in Elderly patientS Starting haemodialysis. *BMJ Open* 2016;6(11):e013081. doi: 10.1136/bmjopen-2016-013081
11. Peden E. Hemodialysis in the Elderly (70yrs & Older) 2016 [Available from: https://ClinicalTrials.gov/show/NCT03065972 accessed december 2020.
12. Murea M, Geary RL, Houston DK, et al. A randomized pilot study to evaluate graft versus fistula vascular access strategy in older patients with advanced kidney disease: results of a feasibility study. *Pilot Feasibility Stud* 2020;6:86. doi: 10.1186/s40814-020-00619-9
13. Yuo TH. Trial of Fistula Versus Graft in Elderly Patients 2018 [Available from: https://ClinicalTrials.gov/show/NCT03668002 accessed december 2020.
14. Rao MK. Comparison of AVF Versus AVG in Elderly Patients Starting Dialysis 2016 [Available from: https://ClinicalTrials.gov/show/NCT02981706 accessed december 2020.
15. Sengoelge G. AUSTrian Randomized Interventional Study on Dialysis Accesses 2019 [Available from: https://ClinicalTrials.gov/show/NCT03764358 accessed december 2020.
16. Dusseux E, Albano L, Fafin C, et al. A simple clinical tool to inform the decision-making process to refer elderly incident dialysis patients for kidney transplant evaluation. *Kidney Int* 2015;88(1):121-9. doi: 10.1038/ki.2015.25
17. Korevaar JC, Jansen MA, Dekker FW, et al. When to initiate dialysis: effect of proposed US guidelines on survival. *Lancet* 2001;358(9287):1046-50. doi: 10.1016/s0140-6736(01)06180-3
18. Ramspek CL, Voskamp PW, van Ittersum FJ, et al. Prediction models for the mortality risk in chronic dialysis patients: a systematic review and independent external validation study. *Clin Epidemiol* 2017;9:451-64. doi: 10.2147/clep.S139748
19. de Goeij MCM, van Diepen M, Jager KJ, et al. Multiple imputation: dealing with missing data. *Nephrology Dialysis Transplantation* 2013;28(10):2415-20. doi: 10.1093/ndt/gft221
20. van Buuren S. Multiple imputation of discrete and continuous data by fully conditional specification. *Statistical Methods in Medical Research* 2007;16(3):219-42. doi: 10.1177/0962280206074463

For peer review only

21. Rubin DB. Multiple imputation for survey nonresponse: Wiley, New York, 1987.
22. Vanholder R, Canaud B, Fluck R, et al. Diagnosis, prevention and treatment of haemodialysis catheter-related bloodstream infections (CRBSI): a position statement of European Renal Best Practice (ERBP). *NDT Plus* 2010;3(3):234-46. doi: 10.1093/ndtplus/sfq041
23. Baskin JL, Reiss U, Wilimas JA, et al. Thrombolytic therapy for central venous catheter occlusion. *Haematologica* 2012;97(5):641-50. doi: 10.3324/haematol.2011.050492
24. Viecelli AK, Tong A, O'Lone E, et al. Report of the Standardized Outcomes in Nephrology-Hemodialysis (SONG-HD) Consensus Workshop on Establishing a Core Outcome Measure for Hemodialysis Vascular Access. *Am J Kidney Dis* 2018;71(5):690-700. doi: 10.1053/j.ajkd.2017.12.003
25. Weisbord SD, Fried LF, Arnold RM, et al. Development of a symptom assessment instrument for chronic hemodialysis patients: the Dialysis Symptom Index. *J Pain Symptom Manage* 2004;27(3):226-40. doi: 10.1016/j.jpainsymman.2003.07.004
26. Kosa SD, Bhola C, Lok CE. Measuring patient satisfaction with vascular access: vascular access questionnaire development and reliability testing. *J Vasc Access* 2015;16(3):200-5. doi: 10.5301/jva.5000339
27. Li B, Cairns JA, Draper H, et al. Estimating Health-State Utility Values in Kidney Transplant Recipients and Waiting-List Patients Using the EQ-5D-5L. *Value Health* 2017;20(7):976-84. doi: 10.1016/j.jval.2017.01.011
28. Beathard GA, Lok CE, Glickman MH, et al. Definitions and End Points for Interventional Studies for Arteriovenous Dialysis Access. *Clin J Am Soc Nephrol* 2018;13(3):501-12. doi: 10.2215/cjn.11531116
29. Al-Balas A, Lee T, Young CJ, et al. The Clinical and Economic Effect of Vascular Access Selection in Patients Initiating Hemodialysis with a Catheter. *J Am Soc Nephrol* 2017;28(12):3679-87. doi: 10.1681/asn.2016060707
30. Gu K, Ng HK, Tang ML, et al. Testing the ratio of two poisson rates. *Biom J* 2008;50(2):283-98. doi: 10.1002/bimj.200710403
31. Nederland Z. Richtlijn voor het uitvoeren van economische evaluaties in de gezondheidszorg., 2015.
32. Bouwmans CA H-vRL, Koopmanschap MA, et al. Manual of the iMTA Medical Consumption Questionnaire (iMCQ). *Rotterdam: iMTA, Erasmus University Rotterdam* 2013
33. Brunelli SM, Van Wyck DB, Njord L, et al. Cluster-Randomized Trial of Devices to Prevent Catheter-Related Bloodstream Infection. *J Am Soc Nephrol* 2018;29(4):1336-43. doi: 10.1681/asn.2017080870
34. Farrington CA, Allon M. Complications of Hemodialysis Catheter Bloodstream Infections: Impact of Infecting Organism. *Am J Nephrol* 2019;50(2):126-32. doi: 10.1159/000501357

Figure legends

Figure 1: Study flow chart. On referral for vascular access creation, patients are screened for eligibility. Patients who meet the inclusion and exclusion criteria and provide informed consent will be randomised and allocated to one of the treatment strategies. Follow-up for the primary and

For peer review only

secondary outcomes starts at the moment of treatment allocation. PROM: Patient-reported outcome measure.

Figure 2: Calibration plot presenting the mortality risk as predicted by the Dusseux risk score and the observed risk in the NECOSAD cohort, such that the 45˚ line indicates perfect agreement between predicted and observed risks.

Figure 3: Kaplan Meier curve comparing the survival probability for the high risk and the low risk group in the NECOSAD cohort.

For peer review only

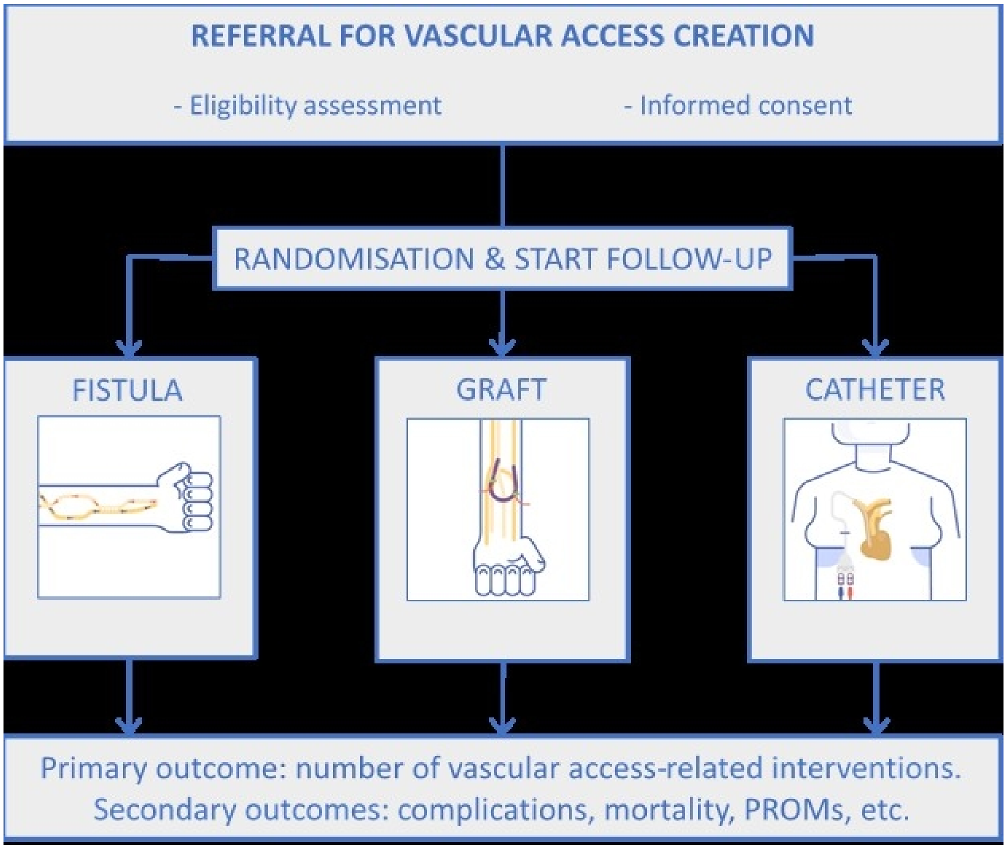

Study flow chart. On referral for vascular access creation, patients are screened for eligibility. Patients who meet the inclusion and exclusion criteria and provide informed consent will be randomised and allocated to one of the treatment strategies. Follow-up for the primary and secondary outcomes starts at the moment of treatment allocation. PROM: Patient-reported outcome measure.

166x139mm (96 x 96 DPI)

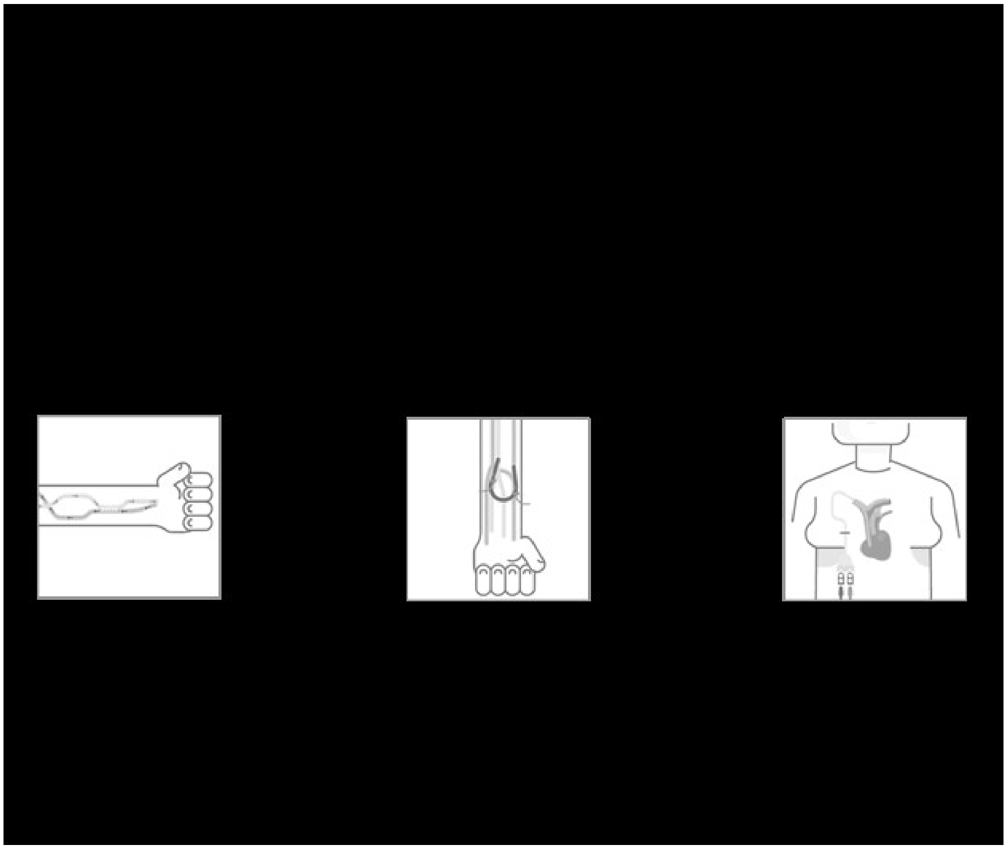

Study flow chart. On referral for vascular access creation, patients are screened for eligibility. Patients who meet the inclusion and exclusion criteria and provide informed consent will be randomised and allocated to one of the treatment strategies. Follow-up for the primary and secondary outcomes starts at the moment of treatment allocation. PROM: Patient-reported outcome measure.

166x139mm (96 x 96 DPI)

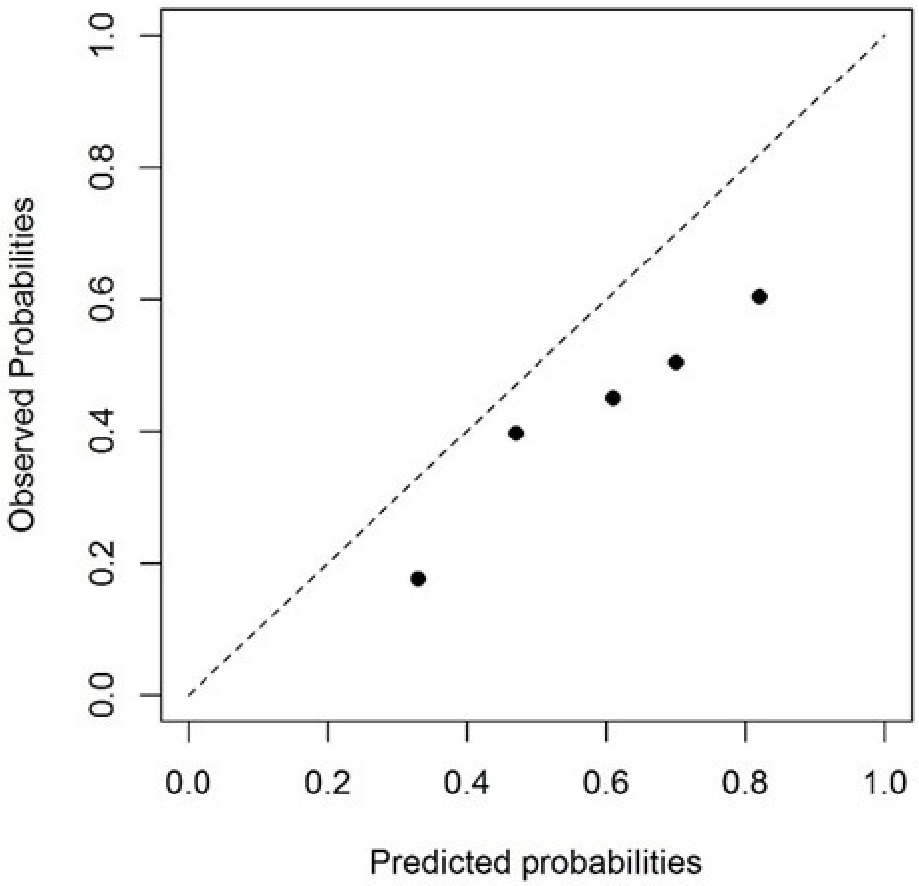

Calibration plot presenting the mortality risk as predicted by the Dusseux risk score and the observed risk in the NECOSAD cohort, such that the 45° line indicates perfect agreement between predicted and observed risks.

85x83mm (150 x 150 DPI)

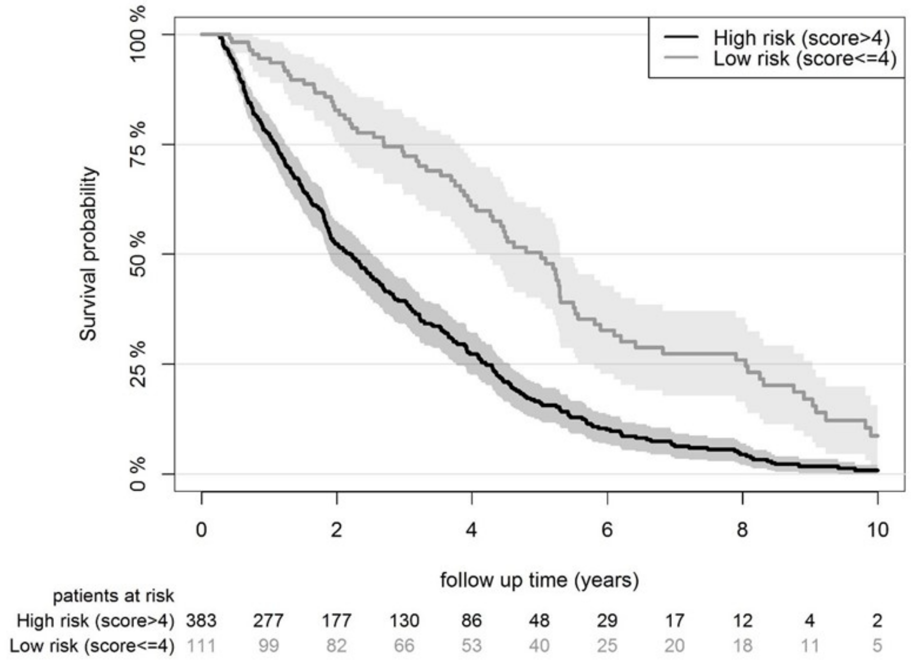

Kaplan Meier curve comparing the survival probability for the high risk and the low risk group in the NECOSAD cohort.

143x100mm (150 x 150 DPI)

Supplementary data 1: The Dusseux score with predictor definitions.(16)

Score item	Score points	Definition given in Dusseux article
Gender		
Male	1	
Female	0	
Age		
70-75	0	
75-80	2	
80-85	5	
≥85	9	
Diabetes		
Yes	2	
No	0	
Ischemic heart disease		history of coronary vascular disease, myocardial infarction, coronary artery bypass surgery, angioplasty, or abnormal angiography
Yes	2	
No	0	
PVD stage III or IV		Leriche-Fontaine classification
No	0	
Stage I or II	0	
Stage III or IV	5	
Cerebrovascular disease		
Yes	1	
No	0	
Chronic heart failure		New York Heart Association stages I–IV
No	0	
Stage I or II	2	
Stage III or IV	4	
Dysrhythmia		defined as atrial fibrillation and in the case of specific treatment with one or more antiarrhythmics, or the presence of a pacemaker or a cardiac implantable defibrillator
Yes	2	
No	0	
Chronic Respiratory disease		Including chronic respiratory failure, defined as a steady-state PaO2 o60mmHg regardless of the level of capnia, a sleep apnea syndrome or requiring oxygen therapy or home ventilatory assistance, and chronic obstructive pulmonary disease, defined as a cough with permanent or recurrent sputum, especially in the morning, 3 months/years for 2 consecutive years
Yes	2	
No	0	
Active malignancy		
Yes	5	
No	0	
Severe behavioural disorder		including dementia, psychosis, or severe neurosis that may affect patient dependence or compliance with treatment
Yes	6	
No	0	
Mobility		
Walks without help	0	
Needs assistance for transfers	4	
Totally dependent for transfers	9	
BMI		
<21	3	
21-25	1	
≥25	0	
CVC at dialysis initiation		
Yes	3	
No	0	

For peer review only

For peer review only

Reporting checklist for protocol of a clinical trial.

Based on the SPIRIT guidelines.

Instructions to authors

Complete this checklist by entering the page numbers from your manuscript where readers will find each of the items listed below.

Your article may not currently address all the items on the checklist. Please modify your text to include the missing information. If you are certain that an item does not apply, please write "n/a" and provide a short explanation.

Upload your completed checklist as an extra file when you submit to a journal.

In your methods section, say that you used the SPIRITreporting guidelines, and cite them as:

Chan A-W, Tetzlaff JM, Gøtzsche PC, Altman DG, Mann H, Berlin J, Dickersin K, Hróbjartsson A, Schulz KF, Parulekar WR, Krleža-Jerić K, Laupacis A, Moher D. SPIRIT 2013 Explanation and Elaboration: Guidance for protocols of clinical trials. BMJ. 2013;346:e7586

		Reporting Item	Page Number
**Administrative information**			
Title	#1	Descriptive title identifying the study design, population, interventions, and, if applicable, trial acronym	1

For peer review only

Trial registration	#2a	Trial identifier and registry name. If not yet registered, name of intended registry	2
Trial registration: data set	#2b	All items from the World Health Organization Trial Registration Data Set	2
Protocol version	#3	Date and version identifier	2
Funding	#4	Sources and types of financial, material, and other support	10
Roles and responsibilities: contributorship	#5a	Names, affiliations, and roles of protocol contributors	10
Roles and responsibilities: sponsor contact information	#5b	Name and contact information for the trial sponsor	1
Roles and responsibilities: sponsor and funder	#5c	Role of study sponsor and funders, if any, in study design; collection, management, analysis, and interpretation of data; writing of the report; and the decision to submit the report for publication, including whether they will have ultimate authority over any of these activities	10
Roles and responsibilities: committees	#5d	Composition, roles, and responsibilities of the coordinating centre, steering committee, endpoint adjudication committee, data management team, and	8

For peer review only

		other individuals or groups overseeing the trial, if applicable (see Item 21a for data monitoring committee)	
**Introduction**			
Background and rationale	#6a	Description of research question and justification for undertaking the trial, including summary of relevant studies (published and unpublished) examining benefits and harms for each intervention	3,4
Background and rationale: choice of comparators	#6b	Explanation for choice of comparators	3,4
Objectives	#7	Specific objectives or hypotheses	4
Trial design	#8	Description of trial design including type of trial (eg, parallel group, crossover, factorial, single group), allocation ratio, and framework (eg, superiority, equivalence, non-inferiority, exploratory)	4
**Methods: Participants, interventions, and outcomes**			
Study setting	#9	Description of study settings (eg, community clinic, academic hospital) and list of countries where data will be collected. Reference to where list of study sites can be obtained	4

For peer review only

Eligibility criteria	#10	Inclusion and exclusion criteria for participants. If applicable, eligibility criteria for study centres and individuals who will perform the interventions (eg, surgeons, psychotherapists)	4,5
Interventions: description	#11a	Interventions for each group with sufficient detail to allow replication, including how and when they will be administered	6,7
Interventions: modifications	#11b	Criteria for discontinuing or modifying allocated interventions for a given trial participant (eg, drug dose change in response to harms, participant request, or improving / worsening disease)	7
Interventions: adherance	#11c	Strategies to improve adherence to intervention protocols, and any procedures for monitoring adherence (eg, drug tablet return; laboratory tests)	7
Interventions: concomitant care	#11d	Relevant concomitant care and interventions that are permitted or prohibited during the trial	N/A
Outcomes	#12	Primary, secondary, and other outcomes, including the specific measurement variable (eg, systolic blood pressure), analysis metric (eg, change from baseline, final value, time to event), method of aggregation (eg, median, proportion), and time point for each outcome. Explanation of the clinical relevance of chosen efficacy and harm outcomes is strongly recommended	7,8

For peer review only

Participant timeline	#13	Time schedule of enrolment, interventions (including any run-ins and washouts), assessments, and visits for participants. A schematic diagram is highly recommended (see Figure)	4,5,9
Sample size	#14	Estimated number of participants needed to achieve study objectives and how it was determined, including clinical and statistical assumptions supporting any sample size calculations	8
Recruitment	#15	Strategies for achieving adequate participant enrolment to reach target sample size	4
**Methods: Assignment of interventions (for controlled trials)**			
Allocation: sequence generation	#16a	Method of generating the allocation sequence (eg, computer-generated random numbers), and list of any factors for stratification. To reduce predictability of a random sequence, details of any planned restriction (eg, blocking) should be provided in a separate document that is unavailable to those who enrol participants or assign interventions	5
Allocation concealment mechanism	#16b	Mechanism of implementing the allocation sequence (eg, central telephone; sequentially numbered, opaque,	5

For peer review only

		sealed envelopes), describing any steps to conceal the sequence until interventions are assigned	
Allocation: implementation	#16c	Who will generate the allocation sequence, who will enrol participants, and who will assign participants to interventions	5
Blinding (masking)	#17a	Who will be blinded after assignment to interventions (eg, trial participants, care providers, outcome assessors, data analysts), and how	4
Blinding (masking): emergency unblinding	#17b	If blinded, circumstances under which unblinding is permissible, and procedure for revealing a participant's allocated intervention during the trial	4
**Methods: Data collection, management, and analysis**			
Data collection plan	#18a	Plans for assessment and collection of outcome, baseline, and other trial data, including any related processes to promote data quality (eg, duplicate measurements, training of assessors) and a description of study instruments (eg, questionnaires, laboratory tests) along with their reliability and validity, if known. Reference to where data collection forms can be found, if not in the protocol	4,8

For peer review only

Data collection plan: retention	#18b	Plans to promote participant retention and complete follow-up, including list of any outcome data to be collected for participants who discontinue or deviate from intervention protocols	9
Data management	#19	Plans for data entry, coding, security, and storage, including any related processes to promote data quality (eg, double data entry; range checks for data values). Reference to where details of data management procedures can be found, if not in the protocol	8
Statistics: outcomes	#20a	Statistical methods for analysing primary and secondary outcomes. Reference to where other details of the statistical analysis plan can be found, if not in the protocol	8,9
Statistics: additional analyses	#20b	Methods for any additional analyses (eg, subgroup and adjusted analyses)	8,9
Statistics: analysis population and missing data	#20c	Definition of analysis population relating to protocol non-adherence (eg, as randomised analysis), and any statistical methods to handle missing data (eg, multiple imputation)	9

Methods: Monitoring

Data monitoring: formal committee	#21a	Composition of data monitoring committee (DMC); summary of its role and reporting structure; statement of whether it is independent from the sponsor and competing interests; and reference to where further	8

For peer review only

		details about its charter can be found, if not in the protocol. Alternatively, an explanation of why a DMC is not needed	
Data monitoring: interim analysis	#21b	Description of any interim analyses and stopping guidelines, including who will have access to these interim results and make the final decision to terminate the trial	8
Harms	#22	Plans for collecting, assessing, reporting, and managing solicited and spontaneously reported adverse events and other unintended effects of trial interventions or trial conduct	8
Auditing	#23	Frequency and procedures for auditing trial conduct, if any, and whether the process will be independent from investigators and the sponsor	N/A
**Ethics and dissemination**			
Research ethics approval	#24	Plans for seeking research ethics committee / institutional review board (REC / IRB) approval	9
Protocol amendments	#25	Plans for communicating important protocol modifications (eg, changes to eligibility criteria, outcomes, analyses) to relevant parties (eg, investigators, REC / IRBs, trial participants, trial registries, journals, regulators)	N/A

For peer review only

Consent or assent	#26a	Who will obtain informed consent or assent from potential trial participants or authorised surrogates, and how (see Item 32)	5
Consent or assent: ancillary studies	#26b	Additional consent provisions for collection and use of participant data and biological specimens in ancillary studies, if applicable	N/A
Confidentiality	#27	How personal information about potential and enrolled participants will be collected, shared, and maintained in order to protect confidentiality before, during, and after the trial	8
Declaration of interests	#28	Financial and other competing interests for principal investigators for the overall trial and each study site	10
Data access	#29	Statement of who will have access to the final trial dataset, and disclosure of contractual agreements that limit such access for investigators	10
Ancillary and post trial care	#30	Provisions, if any, for ancillary and post-trial care, and for compensation to those who suffer harm from trial participation	N/A
Dissemination policy: trial results	#31a	Plans for investigators and sponsor to communicate trial results to participants, healthcare professionals, the public, and other relevant groups (eg, via publication, reporting in results databases, or other data sharing arrangements), including any publication restrictions	10

For peer review only

Dissemination policy: authorship	#31b	Authorship eligibility guidelines and any intended use of professional writers	10
Dissemination policy: reproducible research	#31c	Plans, if any, for granting public access to the full protocol, participant-level dataset, and statistical code	10

Appendices

Informed consent materials	#32	Model consent form and other related documentation given to participants and authorised surrogates	N/A
Biological specimens	#33	Plans for collection, laboratory evaluation, and storage of biological specimens for genetic or molecular analysis in the current trial and for future use in ancillary studies, if applicable	N/A

None The SPIRIT Explanation and Elaboration paper is distributed under the terms of the Creative Commons Attribution License CC-BY-NC. This checklist can be completed online using https://www.goodreports.org/, a tool made by the EQUATOR Network in collaboration with Penelope.ai

For peer review only

BMJ Open

Optimising Access Surgery In Senior haemodialysis patients (OASIS): Study protocol for a multicentre randomised controlled trial

Journal:	*BMJ Open*
Manuscript ID	bmjopen-2021-053108.R1
Article Type:	Protocol
Date Submitted by the Author:	29-Nov-2021
Complete List of Authors:	Heggen, Boudewijn; Maastricht University Medical Centre+, Department of Vascular Surgery<br>Ramspek, Chava; Leiden University Medical Center, Department of Clinical Epidemiology<br>van der Bogt, Koen; Haaglanden Medical Centre, Department of Surgery; Leiden University Medical Center, Department of Surgery<br>de Haan, Michiel; Maastricht University Medical Center, Department of Radiology<br>Hemmelder, Marc; Maastricht University Medical Center, Department of Internal Medicine, division of nephrology<br>Hiligsmann , Mickaël ; Maastricht University, Department of Health Services Research, CAPHRI Care & Public Health Research Institute<br>van Loon, Magda; Maastricht University Medical Center, Department of Vascular Surgery<br>Rotmans, Joris; Leiden University Medical Center, Department of Internal Medicine<br>Tordoir, Jan; Maastricht University Medical Center, Department of Vascular Surgery<br>Dekker, Friedo; Leiden University Medical Centre, Department of Clinical Epidemiology<br>Schurink, Geert-Willem; Maastricht Universitair Medisch Centrum+, Department of Vascular Surgery<br>Snoeijs, Maarten; Maastricht University Medical Center, Department of Vascular Surgery
**Primary Subject Heading**:	Surgery
Secondary Subject Heading:	Renal medicine, Health economics, Geriatric medicine
Keywords:	VASCULAR SURGERY, Vascular surgery < SURGERY, Dialysis < NEPHROLOGY, End stage renal failure < NEPHROLOGY, Adult nephrology < NEPHROLOGY

For peer review only - http://bmjopen.bmj.com/site/about/guidelines.xhtml

Optimising Access Surgery In Senior haemodialysis patients (OASIS): Study protocol for a multicentre randomised controlled trial

Boudewijn DC Heggen[1], Chava L Ramspek[2], Koen EA van der Bogt[3,4], Michiel W de Haan[5], Marc H Hemmelder[6], Mickaël JC Hiligsmann[7], Magda M van Loon[1], Joris I Rotmans[8], Jan HM Tordoir[1], Friedo W Dekker[2], Geert Willem H Schurink[1], Maarten GJ Snoeijs[1], on behalf of the OASIS study group[9].

1 Department of Vascular Surgery, Maastricht University Medical Center, Maastricht, The Netherlands.
2 Department of Clinical Epidemiology, Leiden University Medical Center, Leiden, The Netherlands.
3 Department of Surgery, Haaglanden Medical Center, The Hague, The Netherlands.
4 Department of Surgery, Leiden University Medical Center, Leiden, The Netherlands.
5 Department of Radiology, Maastricht University Medical Center, Maastricht, Netherlands.
6 Department of Internal Medicine, division of nephrology, Maastricht University Medical Center, Maastricht, The Netherlands.
7 Department of Health Services Research, CAPHRI Care & Public Health Research Institute, Maastricht University, Maastricht, The Netherlands.
8 Department of Internal Medicine, Leiden University Medical Center, Leiden, The Netherlands.
9 Members of the OASIS study group are listed at the end of the article.

Corresponding author
Name: Boudewijn DC Heggen
Post address: P.O. box 5800, 6202 AZ, Maastricht, The Netherlands, Maastricht University Medical Center, Department of Vascular Surgery.
Email: boudewijn.heggen@mumc.nl

Word count (main text)
3998

For peer review only

ABSTRACT

Introduction

Current evidence on vascular access strategies for haemodialysis patients is based on observational studies that are at high risk of selection bias. For elderly patients, the autologous arteriovenous fistulas that are typically created in usual care may not be the best option because a significant proportion of fistulas either fail to mature or remain unused. In addition, long-term complications associated with arteriovenous grafts and central venous catheters may be less relevant when considering the limited life expectancy of these patients. Therefore, we designed the OASIS trial to determine the best strategy for vascular access creation in elderly haemodialysis patients.

Methods and analysis

OASIS is a multicentre randomised controlled trial with an equal participant allocation in three treatment arms. Patients aged 70 years or older who are expected to initiate haemodialysis treatment in the next 6 months or who have started haemodialysis urgently with a catheter will be enrolled. To detect and exclude patients with an unusually long life expectancy, a previously published mortality prediction model will be used after external validation. Participants allocated to the usual care arm will be treated according to current guidelines on vascular access creation and will undergo fistula creation. Participants allocated to one of the two intervention arms undergo graft placement or catheter insertion. The primary outcome is the number of access-related interventions required for each patient-year of haemodialysis treatment. We will enrol 195 patients to have sufficient statistical power to detect an absolute decrease of 0.80 interventions per year.

Ethics and dissemination

Because of clinical equipoise, we believe it is justified to randomly allocate elderly patients to the different vascular access strategies. The study was approved by an accredited Medical Ethics Review Committee. The results will be disseminated through peer-reviewed publications and will be implemented in clinical practice guidelines.

Trial registration

Netherlands trial register, NL7933, registered on August 4th, 2019.

Protocol version and date

Version 5, February 25th 2021

Key words

Vascular access, haemodialysis, elderly, arteriovenous fistula, central venous catheter, arteriovenous graft.

ARTICLE SUMMARY

Strengths and limitations of this study

- This is the first prospective trial randomly allocating elderly haemodialysis patients to the three commonly used vascular access strategies.
- By enrolling patients upon referral for vascular access creation, the trial will reflect everyday practice and study patients with pre-dialysis vascular access creation as well as patients who started dialysis urgently with a temporary catheter.
- The primary outcome, i.e. the number of access-related interventions required for each year of haemodialysis treatment, corresponds to the recently proposed core outcome measure for clinical trials in vascular access.
- A previously published mortality prediction score was validated to determine a cut-off point for exclusion of elderly patients with an unusually long life expectancy for whom a fistula is still considered to be the best vascular access.

For peer review only

INTRODUCTION

The haemodialysis population has been growing older and older over the past decades, and the median age of patients starting haemodialysis treatment today is 66 years.[1] These patients need a reliable vascular access that may be provided by autologous arteriovenous fistulas, prosthetic arteriovenous grafts, and central venous catheters. Autologous fistulas are created by surgically anastomosing a superficial vein to an artery in the upper extremity. After creation of the fistula, vascular remodelling will increase venous outflow diameter and blood flow, a process referred to as maturation. Functional fistulas are generally associated with the best long-term outcomes. However, a substantial proportion of fistulas fail to mature and never become functional.[2] Arteriovenous grafts are placed by interposing a prosthetic vascular graft between an artery and a vein in a subcutaneous track in the upper extremity. Grafts do not need time to mature, but are associated with a higher incidence of infection, stenosis, and thrombosis than functional fistulas.[3] Central venous catheters are inserted in the internal jugular vein with a minimally invasive procedure and can be used immediately after placement. However, catheters have been associated with increased bloodstream infections and mortality compared to other vascular access types.[3, 4] Studies comparing these different vascular access strategies typically focus on functional outcomes, and little is known about differences in patient reported outcome measures and health care costs.

Clinical practice guidelines on vascular access have long recommended the creation of autologous arteriovenous fistulas regardless of patient characteristics.[5] However, this recommendation is based solely on observational studies that are at high risk of bias. Patients who have a fistula created prior to starting dialysis generally have more favourable baseline characteristics and a lower mortality risk than other patients.[6] These favourable patient characteristics may account for two-thirds of the survival benefit seen in patients using arteriovenous fistulas for haemodialysis treatment instead of central venous catheters.[7] Further selection bias will occur as patients are analysed according to vascular access use. This tends to favour autologous fistulas since the poor subset of patients with fistulas that have failed to mature will go on to use a different type of vascular access. When primary fistula failures are taken into account, the cumulative patency of fistulas and grafts become comparable.[8] Finally, observational studies based on dialysis registries do not include patients with arteriovenous fistulas created before dialysis initiation that were never used because of patient death or stable renal function.

Uncertainty regarding the best type of vascular access is particularly important for elderly patients starting haemodialysis treatment. In these patients, autologous arteriovenous fistulas have a 40% increased risk of non-maturation and early failure.[2] Moreover, 30% of fistulas created before initiation of dialysis treatment in elderly patients will never be used because of patient death, stabilization of renal function, or choice for conservative treatment as the patient's condition worsens over time.[9] Furthermore, the increase in long-term complications associated with arteriovenous grafts may be less relevant for elderly patients with a limited life expectancy. Finally, elderly patients have a 30% lower risk of complications of central venous catheters, including bloodstream infections.[4, 10] Considering this clinical equipoise, the need for better evidence on vascular access strategies in elderly haemodialysis patients is broadly recognised. Indeed, international experts on vascular access surgery as well as patient representatives have called for a randomised trial comparing vascular access strategies in elderly haemodialysis patients.[5, 11]

We performed a comprehensive search in international trial registers for ongoing and planned randomised trials on vascular access creation in elderly haemodialysis patients. Four ongoing trials were identified that allocate participants to arteriovenous fistula or graft surgery. One trial has a single centre design and plans to enrol 270 haemodialysis patients aged 70 years or older. The participants are followed for two years after vascular access creation for the primary outcomes, which are vascular access use as well as patency.[12] The remaining three trials are pilot studies evaluating the feasibility of the trial design. These pilot studies allocate patients aged 65 years or

For peer review only

older, either at the time of referral for vascular access, at dialysis initiation, or after dialysis initiation with a catheter.[13-15] We also identified two trials allocating participants to fistula creation or catheter insertion. The ACCESS HD feasibility study allocates patients aged 55 years or older who have already commenced haemodialysis treatment with a catheter to either fistula creation or continued catheter use.[11] The AUSTRIA study aims to allocate 220 participants aged 60 years or older to one of the two treatment arms, with access-related complications being the primary outcome measure.[16] Although these ongoing studies will surely provide valuable information, none of the trials cover the full spectrum of vascular access strategies in elderly haemodialysis patients.

The aim of the OASIS trial is to determine the best vascular access strategy for elderly haemodialysis patients. We hypothesize that arteriovenous grafts and central venous catheters are superior to autologous arteriovenous fistula for elderly patients starting haemodialysis treatment and result in reduced access-related intervention rates, greater patient satisfaction, and lower health care costs.

METHODS AND ANALYSIS

Study design

OASIS is a parallel group, multicentre, randomised controlled trial, with a superiority framework and a 1:1:1 individual participant treatment allocation ratio over 3 study arms. Given the nature of the interventions, blinding health care providers, participants, or outcome assessors for treatment assignment is not possible. However, data analysis will be performed without knowledge of treatment assignment by coding the treatment arms and unlocking the code only after statistical analysis is completed. Some 17 hospitals, together treating approximately 50% of all haemodialysis patients in the Netherlands, are expected to participate.

Study population

Upon referral for permanent vascular access creation, all patients will have a preoperative duplex ultrasound assessment of the blood vessels of the upper extremities. This preoperative workup may be supplemented by central venous phlebography and arterial imaging as considered appropriate by the local vascular access team. All potential participants will receive oral and written information about the trial by the local investigators and are registered in a screening log.

Inclusion criteria:

1. Age 70 years or older;
2. End-stage renal disease with unlikely recovery of kidney function according to the attending nephrologist;
3. Haemodialysis is the intended long-term modality of treatment for end-stage renal disease;
4. Fit for any of the proposed vascular access interventions as determined by the local multidisciplinary vascular access team;
5. a. Expected to start haemodialysis treatment within 6 months at the time of treatment assignment; or
   b. Treated with haemodialysis for 6 months or less at the time of treatment assignment using a tunnelled or non-tunnelled central venous catheter for vascular access;
6. Planning to remain in one of participating dialysis centres for at least 1 year;
7. Suitable vascular anatomy for all types of vascular access based on duplex ultrasound of the arms, defined as:[5]
   - at least one suitable configuration for an arteriovenous fistula using minimal arterial and venous diameters of 2mm for radiocephalic fistulas and 3mm for brachiocephalic and brachiobasilic fistulas;
   - at least one suitable configuration for an arteriovenous graft using minimal arterial and venous diameters of 3mm and 4mm, respectively; and
   - at least one open internal jugular vein for a central venous catheter.

For peer review only

Exclusion criteria:

1. Patent arteriovenous fistula or graft already in place;
2. Prior unsuccessful arteriovenous fistula or graft vascular access surgery;
3. Kidney transplantation planned within 6 months;
4. Metastatic malignancies or other condition associated with a life expectancy of <6 months, in the opinion of the attending nephrologist;
5. Unable to provide informed consent;
6. Dusseux risk score ≤ 4, indicating an unusually long life expectancy.[17]

Patients will be enrolled by local investigators if they meet the eligibility criteria and provide informed consent. Local investigators will be informed of treatment assignment through a web-based service, after which vascular access creation is planned according to usual clinical care for the allocated vascular access type at the study site (Figure 1). The treatment allocation sequence is generated by an independent data management centre using a computer-based random number producing algorithm. Block randomisation is implemented with randomly varying block sizes of 3 and 6, and randomisation will be stratified by treatment centre. Follow-up starts immediately after treatment allocation.

External validation of a mortality prediction model

Elderly haemodialysis patients form a heterogeneous population in terms of comorbidities and life expectancy. The majority of elderly haemodialysis patients have multiple comorbidities and a relatively short life expectancy making the long-term performance of the vascular access less relevant. However, for the small group of elderly patients with an unusually long life expectancy, autologous arteriovenous fistulas are considered to be the best vascular access given their low complication rates after maturation. To identify this subset of elderly patients that should be excluded from randomisation, we performed an external validation of the mortality prediction score developed by Dusseux et al.[17]. This prediction score contains items readily identified from the medical history and can be completed before initiation of haemodialysis treatment.

The mortality prediction score was validated in the Netherlands Cooperative Study on the Adequacy of Dialysis (NECOSAD).[18, 19] In this multicentre observational cohort study, adult patients starting dialysis treatment between 1997 and 2007 in the Netherlands were prospectively followed until February 1st, 2015. For the current validation, we restricted the sample to haemodialysis patients aged 70 years and older. This resulted in 494 patients, 411 of whom died during follow-up with a median survival of 2.3 years (interquartile range: 1.1-4.4 years). Overall, baseline characteristics in the validation cohort were comparable to those in the Dusseux cohort (table 1).[17] Missing data were assumed to be largely missing at random. Therefore, 10-fold multiple imputation with fully conditional specification was performed using the R package 'mice'. All variables of the mortality prediction score and the outcome were included in the imputation model.[20, 21]

Each individual's risk score was calculated with the original point system as presented by Dusseux (supplementary data 1). Risk scores ranged from 0 to 28, with a higher score corresponding to a higher risk of mortality. From the individual risk scores, the discrimination for mortality within 2 years was assessed by c-statistics that were pooled over the ten imputation datasets according to Rubin's Rules.[22] The Dusseux mortality risk score had a c-statistic of 0.68 (95% CI: 0.63-0.73) in the NECOSAD validation cohort. Furthermore, the cohort was divided into 5 groups with increasing mortality risk. A calibration plot of predicted versus observed mortality risk was computed. Calibration was moderate as the model tended to overpredict the mortality risk (53% average predicted risk and 38% average observed risk; Figure 2). Finally, we determined a sensible cut-off point for identifying a subset of patients with an unusually long life expectancy. A cut-off between a score of ≤4 and ≥5 was chosen to separate patients with unusually good outcomes (consisting of 22% of patients in the validation cohort with a median survival of 5.0 years (95% CI: 4.3-5.4)) from elderly

For peer review only

haemodialysis patients with a poor life expectancy (consisting of the remaining 78% of patients in the validation cohort with a median survival of 2.2 years (95% CI: 1.9-2.5); Figure 3).

	**NECOSAD cohort n=494**	**Dusseux cohort n=8955**
Gender (male)	61 %	60 %
Age	76 (73 – 79)	78 (74 – 82)
Diabetes	25 %	38 %
Ischemic heart disease[1]	31 %	14 %
Peripheral vascular disease	24 %	27%
Cerebrovascular disease	14 %	12 %
Congestive heart failure	19 %	34 %
Dysrhythmia[2]	22 %	25 %
Respiratory disease[3]	12%	13 %
Active malignancy	4 %	11 %
Psychiatric disorder[4]	3 %	4 %
Mobility[5, 6]		
- Walks without help	64 %	69 %
- Needs assistance for transfers	32 %	22 %
- Totally dependent for transfers	4 %	9 %
BMI (Median)[7]		
- < 21 kg/m^2	13 %	18 %
- 22 – 25 kg kg/m^2	43 %	35 %
- 25 – 30 kg/m^2	33 %	33 %
- > 30 kg/m^2	10 %	14 %
CVC at dialysis initiation	23 %	42 %

Table 1: Baseline characteristics of the NECOSAD validation cohort compared to the Dusseux derivation cohort.[17] 1: Ischemic heart disease was defined as a history of angina pectoris or myocardial infarction. 2: Vitamin K antagonist use was used as a proxy for cardiac dysrhythmia. 3: Respiratory disease was defined as the need to take pulmonary medication on a daily basis. 4: Psychiatric disorder was defined as dementia, depression, or other psychiatric disease. 5: The Karnofsky score was used as a proxy for mobility, where a score of >60 was considered as 'walks without help', a score of 50-60 as 'needs assistance for transfers', and a score <50 as 'totally dependent for transfers'. 6: 32% of the data concerning mobility was missing in the Dusseux cohort. 7: 29% of the data concerning BMI was missing in the Dusseux cohort.

Interventions

The surgical strategies in the usual care and intervention groups are part of standard clinical care. Because we aim to compare the different surgical strategies for vascular access creation in everyday practice, we will not interfere with surgical protocols at the study sites. However, we expect the study sites to follow current best practice and guideline recommendations.[5]

Autologous arteriovenous fistulas

Fistulas are preferably created at the most distal site with adequate blood vessels, in the non-dominant arm. It is recommended to create the fistula 3 to 6 months before the expected start of haemodialysis treatment. In case of fistula failure, a new arteriovenous fistula may be created at the next available site.

For peer review only

Prosthetic arteriovenous grafts

Patients who are allocated to the graft strategy will have a commercially available prosthetic vascular graft implanted for haemodialysis access. It is recommended to place the arteriovenous graft two weeks before the expected start of haemodialysis treatment with antibiotic prophylaxis. Placement of an early-cannulation graft is recommended for patients who require more urgent start of haemodialysis to avoid the use of a temporary central venous catheter. In case of graft failure despite thrombectomy, a new graft may be placed.

Central venous catheters

Patients who are allocated to the catheter strategy will have a dialysis catheter inserted, unless they have already started haemodialysis with a tunnelled catheter. We recommend placing a tunnelled catheter just before the start of haemodialysis treatment. We advise to follow published guidelines for the prevention and treatment of catheter infections.[23] In case of catheter dysfunction despite local thrombolytic agents, the catheter can be exchanged.[24]

For the arteriovenous fistula and graft group, surveillance and pre-emptive intervention for stenosis will take place according to usual practice at the study sites. In case of failure, both endovascular and surgical interventions can be undertaken to restore patency. If the assigned vascular access strategy proves not to be feasible, patients will crossover to one of the other treatment groups after discussion of the case with the principal investigator. Use of a temporary central venous catheter is not considered as a treatment group crossover.

Primary study outcome

The primary study outcome is the number of access-related interventions required for each person-year of haemodialysis treatment. To analyse this, we will divide the total number of interventions during follow-up by the total number of haemodialysis years. The primary outcome corresponds to the proposed core outcome measure for haemodialysis vascular access.[25] This outcome measure includes all percutaneous access interventions, surgical access procedures, and catheter interventions from randomisation until the end of the study period or death. The outcome measure specifically includes interventions before dialysis initiation and after dialysis cessation in the occasional patient who stops haemodialysis treatment after kidney transplantation, peritoneal dialysis, recovery of renal function, or refusal of further haemodialysis. These vascular access interventions before dialysis initiation and after dialysis cessation are included as they are part of the standard care that this trial evaluates. Patients who discontinue haemodialysis treatment will therefore remain in the trial. Access-related complications that are resolved using conservative or pharmacological treatment are not counted as interventions.

Secondary study outcomes

Complications

Access-related complications requiring pharmacological, surgical, or endovascular interventions will be registered from randomisation until the end of the study period or death. Furthermore, the number of days admitted to hospital or visiting out-patient clinics for any reason and for vascular access-related reasons per person-year will be registered. Finally, all-cause mortality from randomisation to the end of the study period will be registered.

Patient-reported outcome measures (PROMs)

1. Short-Form Health Survey (SF-12) and the Dialysis Symptom Index (DSI) measure generic health-related quality of life as well as disease-specific symptom burden.[26]
2. The Short-Form Vascular Access Questionnaire (SF-VAQ) measures haemodialysis patients' satisfaction with their vascular access.[27]
3. The 5-level EuroQol 5-dimensional questionnaire (EQ-5D-5L) measures health-state utility values.[28]

For peer review only

The SF-VAQ will be administered on a monthly basis, whereas the SF-12, DSI and EQ-5D-5L will be administered at 3-month intervals. To lower their burden, participants that have started haemodialysis treatment will receive the questionnaires during these treatments and get assistance from a nurse. The questionnaires will be administered from randomisation until the one year after study or dialysis initiation, whichever is later.

Other outcome measures will be registered for exploratory analyses and are defined according to the ESVS guidelines on vascular access:[5]

1. Primary, assisted primary, secondary, and functional patency of vascular access.
2. Time until vascular access maturation (definition according to American Society of Nephrology).[29]
3. Time until functional vascular access.
4. The number of haemodialysis sessions with inadequate haemodialysis dose (Kt/V) and with cannulation difficulties or failure. Cannulation difficulties or failures will be recorded with the use of monthly questionnaires asking dialysis nurses whether more than one cannulation attempt was required, whether two-needle cannulation was impossible or if cannulation was painful.

Quality and safety

Data is entered on electronic case report forms by trained investigators using numerical codes that cannot be traced to the individual subjects. The subject identification code list is kept by the principle investigator at each study site. Any serious adverse events that may occur will be reported to the principle investigator without undue delay after obtaining knowledge of the events. To evaluate differences in serious adverse events and mortality between treatment arms, an interim safety analysis will be performed and presented to a Data Safety Monitoring Board every six months. Significant differences on safety outcomes may lead to discontinuation of the trial. Furthermore, an independent monitor will be appointed to assess trial processes and data.

Sample size calculation

Sample size was estimated for the primary study outcome of access-related intervention rates. Since two intervention groups will be compared to the usual care group, we used Bonferroni correction to account for multiple comparisons, which resulted in an alpha of 0.025. The average number of interventions per year for patients receiving an autologous arteriovenous fistula has previously been reported at 2.48 interventions per year.[30] The clinically relevant effect size was considered to be 1 percutaneous intervention per year and 0.5 surgical interventions per year. Since these interventions occur at a ratio of 1:0.7,[30] a reduction of the overall intervention rate by 0.80 interventions per year was used as the clinically relevant effect for sample size calculations.

With these assumptions, a total number of 195 patients (65 patients in each treatment arm) with one year of follow-up achieves 80% power to detect a 0.80 decrease in the number of access-related interventions per person-year of haemodialysis treatment between the intervention groups and the usual care group using a two-sided, large-samples z-test of the Poisson event-rate difference at a significance level of 0.025.[31]

Since the study includes patients with a limited life expectancy, a substantial proportion of participants is expected to die before contributing one year of follow-up. To compensate for the resulting loss of statistical power, the trial has a variable follow-up with trial closeout for all patients at the time when the last patient enrolled has one year of follow-up. Since participant recruitment is expected to take 2 years, the resulting additional follow-up time will more than compensate for the loss of follow-up due to patient mortality in the first year of the study period.

Data analysis plan

For peer review only

The access-related intervention rates will be analysed using a general linear model with Poisson distribution and identity link, and with time as off-set variable. Both intervention (i.e. the prosthetic arteriovenous graft and central venous catheter) groups will be compared to the usual care (autologous arteriovenous fistula) group. Non-adherence to the allocated treatment group is expected as patients cross over to another surgical strategy for vascular access when the randomised strategy is no longer feasible. The primary analysis will be on the intention to treat population. Exploratory on-treatment analyses will be performed as well. A subgroup analysis will be performed for patients who had already started haemodialysis using a catheter before inclusion.

Formal statistical comparisons will be made for the following secondary study outcomes:
1. SF-12 / DSI and SF-VAQ summary scores using generalised estimating equations.
2. The number of access-related serious adverse events per person-year using general linear models with Poisson distribution, and mortality using Cox regression.
3. The number of days admitted to hospital or visiting out-patient clinics for any reason per person-year using student t-tests.

Primary outcome data are not expected to be missing, as interventions on vascular access will be reported in the patients' medical files. We expect little to no loss to follow-up since study participants will be observed three times per week in the dialysis unit and the trial requires no additional study visits. Patient-reported outcome measures will be analysed using generalised estimating equations that allow for missing data. Other missing data will be handled by using 10 imputation cycles with regression methods, performing standard analyses for each imputation cycle, and considering the variability across the imputation cycles in the final analysis.

Economic analysis

A cost effectiveness analysis and a budget impact analysis comparing the different treatment strategies will be performed by collecting financial data from randomisation until the one year after study or dialysis initiation, whichever is later. The economic analyses will be done according to Dutch national guidelines and have a time horizon of 2 years that corresponds to the median life expectancy of the trial participants.[32]

For the cost effectiveness analysis, we will consider both healthcare sector costs and costs for patients and their families. Due to the age of our study population, absenteeism in paid work and production losses in the domestic sphere will not be considered. The relevant cost factors for the healthcare sector will be derived from hospital and pharmacy registration systems at the individual participant level. Costs to patients and families will be measured using a study-specific adaptation of the Medical Consumption Questionnaire developed by the Institute for Medical Technology Assessment at 3-month intervals.[33] After measurement of the relevant cost factors, monetary values will be assigned.

The budget impact analysis will be performed to estimate the financial consequences for the Dutch national health care budget for the different vascular access strategies in elderly haemodialysis patients. The perspectives that will be included in the analysis are the wider societal perspective including productivity losses, the narrower perspective of the public, and the perspective of health care providers and health insurance companies. Several scenarios will be included to assess the impact of different reimbursement options.

Patient and public involvement

The Dutch Renal Patients Society (NVN) was involved in the design of OASIS. We consulted patient representatives on the feasibility of randomisation into the three treatment arms and on the choice of the primary outcome measure. Patient representatives also advised on the frequency and duration of patient-reported outcome measurements and approved the patient information letter.

For peer review only

ETHICS AND DISSEMINATION

Ethics

The study will be conducted according to the principles of the Declaration of Helsinki (Brazil, 2013) and the Medical Research Involving Human Subjects Act (WMO). The protocol was approved by an accredited Medical Ethics Review Committee in November 2019. Some physicians may consider it unethical to use central venous catheters in haemodialysis patients who are candidates for autologous arteriovenous fistula creation, considering the increased mortality and infection rate associated with catheter use. However, there is serious doubt whether a causal relationship between catheter use and mortality exists. The association between catheter use and mortality is derived from observational studies that are profoundly influenced by selection bias. Two examples of this are found in recent studies demonstrating that patient selection for fistula placement, even when they are dialysed with a catheter instead, explains at least two thirds of the mortality benefit observed in patients with a fistula.[6, 7] Furthermore, the majority of the excess mortality observed in patients who use a catheter is felt to be attributable to catheter-related bloodstream infections. However, during the run-in period of a multicentre randomised controlled trial, the incidence of catheter-related bloodstream infections was only 1.0 per 1000 catheter days.[34] In elderly patients, the risk of infection is even lower than in the general haemodialysis population.[9] In a large cohort of dialysis patients with catheter-related bloodstream infections, only 1% of these infections were fatal.[35] Therefore, the observed survival benefit with fistulas can impossibly be explained by avoidance of access-related infections. Moreover, there is clinical equipoise in elderly haemodialysis patients because the balance between risks and benefits of the different types of vascular access is different in this population. Any increase in access-related complications associated with long-term use of grafts and catheters may be less relevant for elderly patients with an expected median survival of 2.2 years. Finally, our study has been designed to minimise the potential risk for participants by scheduling frequent safety analyses, by allowing treatment crossover in case of access complications, and by excluding elderly patients with an unusually long life expectancy.

Dissemination

The OASIS trial is expected to lead to new insights in vascular access surgery for elderly haemodialysis patients. After completion of the trial, the results will be disseminated among vascular surgeons, nephrologists, interventional radiologists, and dialysis nurses through international peer-reviewed publications in medical journals and presentations at scientific meetings.

After the trial results have been published, its findings should be implemented into clinical practice. To identify possible facilitators and obstacles for implementation, OASIS will include a process evaluation. We will register the reasons for patients not participating in the randomised trial in a screening log and we will study ineligible elderly haemodialysis patients in a parallel observational cohort study. Patient characteristics and outcomes of the randomised trial and the observational cohort will be compared to evaluate the generalisability of the trial. Furthermore, we will register study protocol violations during the enrolment and follow-up phase and we will investigate any protocol violations, such as non-adherence to the assigned treatment, by interviewing the treating physician. This process evaluation will refine our implementation plan with strategies tailored to the specific clinical circumstances.

Author contributions

BDCH drafted the manuscript. MGJS designed the study and supervised the writing of the manuscript. CLR and FWD performed the external validation of the mortality prediction score. KEAB, MWH, MHH, MJCH, MML, JIR, JHMT and GWHS provided significant intellectual input in the development of the study protocol. All authors read and approved the final manuscript.

Competing interests

For peer review only

None declared.

Funding statement

This study was funded by Leading the Change (ZonMw project number 80-85009-98-2004). The funder has no role in the design and management of the trial, nor in the data collection and analysis or interpretation and publication of the results.

Data statement

After completion of the trial, data will be made available for further research and verification and will therefore be documented and saved in an accessible manner that is in accordance with the laws and regulations concerning privacy sensitive data.

Group information

Members of the OASIS study group: Marijke J Molegraaf (Department of Vascular Surgery, Isala, Zwolle, The Netherlands); Philippe WM Cuypers (Department of Vascular Surgery, Catharina Ziekenhuis, Eindhoven, The Netherlands). Denise Nio (Department of Vascular Surgery, Spaarne Gasthuis, Haarlem, The Netherlands); Cora H Arts (Department of Vascular Surgery, Medisch Centrum Leeuwarden, Leeuwarden, The Netherlands); Jacobien C Verhave (Department of Internal Medicine, Rijnstate, Arnhem, The Netherlands); Çagdas Ünlü (Department of Vascular Surgery, Noordwest Ziekenhuisgroep, Alkmaar, The Netherlands); Patrick WHE Vriens (Department of Vascular Surgery, Elisabeth-TweeSteden Ziekenhuis, Tilburg, The Netherlands); Goos D Laverman (Department of Internal Medicine, Ziekenhuisgroep Twente, Almelo, The Netherlands); Aron S Bode (Department of Vascular Surgery, Canisius Wilhelmina Ziekenhuis, Nijmegen, The Netherlands); Marcel C Weijmer (Department of Internal Medicine, OLVG, Amsterdam, The Netherlands); Bas Govaert (Department of Vascular Surgery, Máxima Medisch Centrum, Veldhoven, The Netherlands); Aaltje Y Adema (Department of Internal Medicine, Medisch Centrum Leeuwarden, Leeuwarden, The Netherlands); Gijs MJM Welten (Department of Vascular Surgery, Franciscus Gasthuis & Vlietland, Rotterdam, the Netherlands); Michel MPJ Reijnen (Department of Vascular Surgery, Rijnstate, Arnhem, The Netherlands); Marc JH Groeneveld (Department of Internal Medicine, Haaglanden Medisch Centrum, The Hague, The Netherlands); Hans S Brink (Department of Internal Medicine, Medisch Spectrum Twente, Enschede, The Netherlands); Jeroen B van der Net (Department of Internal Medicine, Albert Schweitzer Ziekenhuis, Dordrecht, The Netherlands); Arno Kuijper (Department of Internal Medicine, Máxima Medisch Centrum, Veldhoven, The Netherlands); Hilde PE Peters (Department of Internal Medicine, Isala, Zwolle, The Netherlands); Maarten A Lijkwan (Department of Vascular Surgery, Albert Schweitzer Ziekenhuis, Dordrecht, The Netherlands); Edith M Willigendael (Department of Vascular Surgery, Medisch Spectrum Twente, Enschede, The Netherlands); Roos C van Nieuwenhuizen (Department of Vascular Surgery, OLVG, Amsterdam, The Netherlands); Rombout R Kruse (Department of Vascular Surgery, Ziekenhuisgroep Twente, Almelo, The Netherlands);); Yvonne C Schrama (Department of Internal Medicine, Franciscus Gasthuis & Vlietland, Rotterdam, the Netherlands); Karina A de Groot (Department of Internal Medicine, Noordwest Ziekenhuisgroep, Alkmaar, The Netherlands); Karima Farhat (Department of Internal Medicine, Spaarne Gasthuis, Haarlem/Hoofddorp, The Netherlands).

Supplementary materials

- Supplementary data 1: Point system as presented by Dusseux[17]

References

1. Kramer A, Boenink R, Noordzij M, et al. The ERA-EDTA Registry Annual Report 2017: a summary. *Clin Kidney J* 2020;13(4):693-709. doi: 10.1093/ckj/sfaa048

For peer review only

2. Bylsma LC, Gage SM, Reichert H, et al. Arteriovenous Fistulae for Haemodialysis: A Systematic Review and Meta-analysis of Efficacy and Safety Outcomes. *Eur J Vasc Endovasc Surg* 2017;54(4):513-22. doi: 10.1016/j.ejvs.2017.06.024
3. Almasri J, Alsawas M, Mainou M, et al. Outcomes of vascular access for hemodialysis: A systematic review and meta-analysis. *J Vasc Surg* 2016;64(1):236-43. doi: 10.1016/j.jvs.2016.01.053
4. Poinen K, Quinn RR, Clarke A, et al. Complications From Tunneled Hemodialysis Catheters: A Canadian Observational Cohort Study. *Am J Kidney Dis* 2019;73(4):467-75. doi: 10.1053/j.ajkd.2018.10.014
5. Schmidli J, Widmer MK, Basile C, et al. Editor's Choice - Vascular Access: 2018 Clinical Practice Guidelines of the European Society for Vascular Surgery (ESVS). *Eur J Vasc Endovasc Surg* 2018;55(6):757-818. doi: 10.1016/j.ejvs.2018.02.001
6. Quinn RR, Oliver MJ, Devoe D, et al. The Effect of Predialysis Fistula Attempt on Risk of All-Cause and Access-Related Death. *J Am Soc Nephrol* 2017;28(2):613-20. doi: 10.1681/asn.2016020151
7. Brown RS, Patibandla BK, Goldfarb-Rumyantzev AS. The Survival Benefit of "Fistula First, Catheter Last" in Hemodialysis Is Primarily Due to Patient Factors. *J Am Soc Nephrol* 2017;28(2):645-52. doi: 10.1681/asn.2016010019
8. Lok CE, Sontrop JM, Tomlinson G, et al. Cumulative patency of contemporary fistulas versus grafts (2000-2010). *Clin J Am Soc Nephrol* 2013;8(5):810-8. doi: 10.2215/cjn.00730112
9. Lee T, Thamer M, Zhang Y, et al. Outcomes of Elderly Patients after Predialysis Vascular Access Creation. *J Am Soc Nephrol* 2015;26(12):3133-40. doi: 10.1681/ASN.2014090938
10. Murea M, James KM, Russell GB, et al. Risk of catheter-related bloodstream infection in elderly patients on hemodialysis. *Clin J Am Soc Nephrol* 2014;9(4):764-70. doi: 10.2215/CJN.07710713
11. Quinn R, Ravani P. ACCESS HD pilot: A randomised feasibility trial Comparing Catheters with fistulas in Elderly patientS Starting haemodialysis. *BMJ Open* 2016;6(11):e013081. doi: 10.1136/bmjopen-2016-013081
12. Peden E. Hemodialysis in the Elderly (70yrs & Older) 2016 [Available from: https://ClinicalTrials.gov/show/NCT03065972 accessed december 2020.
13. Murea M, Geary RL, Houston DK, et al. A randomized pilot study to evaluate graft versus fistula vascular access strategy in older patients with advanced kidney disease: results of a feasibility study. *Pilot Feasibility Stud* 2020;6:86. doi: 10.1186/s40814-020-00619-9
14. Yuo TH. Trial of Fistula Versus Graft in Elderly Patients 2018 [Available from: https://ClinicalTrials.gov/show/NCT03668002 accessed december 2020.
15. Rao MK. Comparison of AVF Versus AVG in Elderly Patients Starting Dialysis 2016 [Available from: https://ClinicalTrials.gov/show/NCT02981706 accessed december 2020.
16. Sengoelge G. AUSTrian Randomized Interventional Study on Dialysis Accesses 2019 [Available from: https://ClinicalTrials.gov/show/NCT03764358 accessed december 2020.
17. Dusseux E, Albano L, Fafin C, et al. A simple clinical tool to inform the decision-making process to refer elderly incident dialysis patients for kidney transplant evaluation. *Kidney Int* 2015;88(1):121-9. doi: 10.1038/ki.2015.25

For peer review only

18. Korevaar JC, Jansen MA, Dekker FW, et al. When to initiate dialysis: effect of proposed US guidelines on survival. *Lancet* 2001;358(9287):1046-50. doi: 10.1016/s0140-6736(01)06180-3
19. Ramspek CL, Voskamp PW, van Ittersum FJ, et al. Prediction models for the mortality risk in chronic dialysis patients: a systematic review and independent external validation study. *Clin Epidemiol* 2017;9:451-64. doi: 10.2147/clep.S139748
20. de Goeij MCM, van Diepen M, Jager KJ, et al. Multiple imputation: dealing with missing data. *Nephrology Dialysis Transplantation* 2013;28(10):2415-20. doi: 10.1093/ndt/gft221
21. van Buuren S. Multiple imputation of discrete and continuous data by fully conditional specification. *Statistical Methods in Medical Research* 2007;16(3):219-42. doi: 10.1177/0962280206074463
22. Rubin DB. Multiple imputation for survey nonresponse: Wiley, New York, 1987.
23. Vanholder R, Canaud B, Fluck R, et al. Diagnosis, prevention and treatment of haemodialysis catheter-related bloodstream infections (CRBSI): a position statement of European Renal Best Practice (ERBP). *NDT Plus* 2010;3(3):234-46. doi: 10.1093/ndtplus/sfq041
24. Baskin JL, Reiss U, Wilimas JA, et al. Thrombolytic therapy for central venous catheter occlusion. *Haematologica* 2012;97(5):641-50. doi: 10.3324/haematol.2011.050492
25. Viecelli AK, Tong A, O'Lone E, et al. Report of the Standardized Outcomes in Nephrology-Hemodialysis (SONG-HD) Consensus Workshop on Establishing a Core Outcome Measure for Hemodialysis Vascular Access. *Am J Kidney Dis* 2018;71(5):690-700. doi: 10.1053/j.ajkd.2017.12.003
26. Weisbord SD, Fried LF, Arnold RM, et al. Development of a symptom assessment instrument for chronic hemodialysis patients: the Dialysis Symptom Index. *J Pain Symptom Manage* 2004;27(3):226-40. doi: 10.1016/j.jpainsymman.2003.07.004
27. Kosa SD, Bhola C, Lok CE. Measuring patient satisfaction with vascular access: vascular access questionnaire development and reliability testing. *J Vasc Access* 2015;16(3):200-5. doi: 10.5301/jva.5000339
28. Li B, Cairns JA, Draper H, et al. Estimating Health-State Utility Values in Kidney Transplant Recipients and Waiting-List Patients Using the EQ-5D-5L. *Value Health* 2017;20(7):976-84. doi: 10.1016/j.jval.2017.01.011
29. Beathard GA, Lok CE, Glickman MH, et al. Definitions and End Points for Interventional Studies for Arteriovenous Dialysis Access. *Clin J Am Soc Nephrol* 2018;13(3):501-12. doi: 10.2215/cjn.11531116
30. Al-Balas A, Lee T, Young CJ, et al. The Clinical and Economic Effect of Vascular Access Selection in Patients Initiating Hemodialysis with a Catheter. *J Am Soc Nephrol* 2017;28(12):3679-87. doi: 10.1681/asn.2016060707
31. Gu K, Ng HK, Tang ML, et al. Testing the ratio of two poisson rates. *Biom J* 2008;50(2):283-98. doi: 10.1002/bimj.200710403
32. Nederland Z. Richtlijn voor het uitvoeren van economische evaluaties in de gezondheidszorg., 2015.
33. Bouwmans CA H-vRL, Koopmanschap MA, et al. Manual of the iMTA Medical Consumption Questionnaire (iMCQ). *Rotterdam: iMTA, Erasmus University Rotterdam* 2013

For peer review only

34. Brunelli SM, Van Wyck DB, Njord L, et al. Cluster-Randomized Trial of Devices to Prevent Catheter-Related Bloodstream Infection. *J Am Soc Nephrol* 2018;29(4):1336-43. doi: 10.1681/asn.2017080870
35. Farrington CA, Allon M. Complications of Hemodialysis Catheter Bloodstream Infections: Impact of Infecting Organism. *Am J Nephrol* 2019;50(2):126-32. doi: 10.1159/000501357

Figure legends

Figure 1: Study flow chart. On referral for vascular access creation, patients are screened for eligibility. Patients who meet the inclusion and exclusion criteria and provide informed consent will be randomised and allocated to one of the treatment strategies. Follow-up for the primary and secondary outcomes starts at the moment of treatment allocation. PROM: Patient-reported outcome measure.

Figure 2: Calibration plot presenting the mortality risk as predicted by the Dusseux risk score and the observed risk in the NECOSAD cohort, such that the 45˚ line indicates perfect agreement between predicted and observed risks.

Figure 3: Kaplan Meier curve comparing the survival probability for the high risk and the low risk group in the NECOSAD cohort.

For peer review only

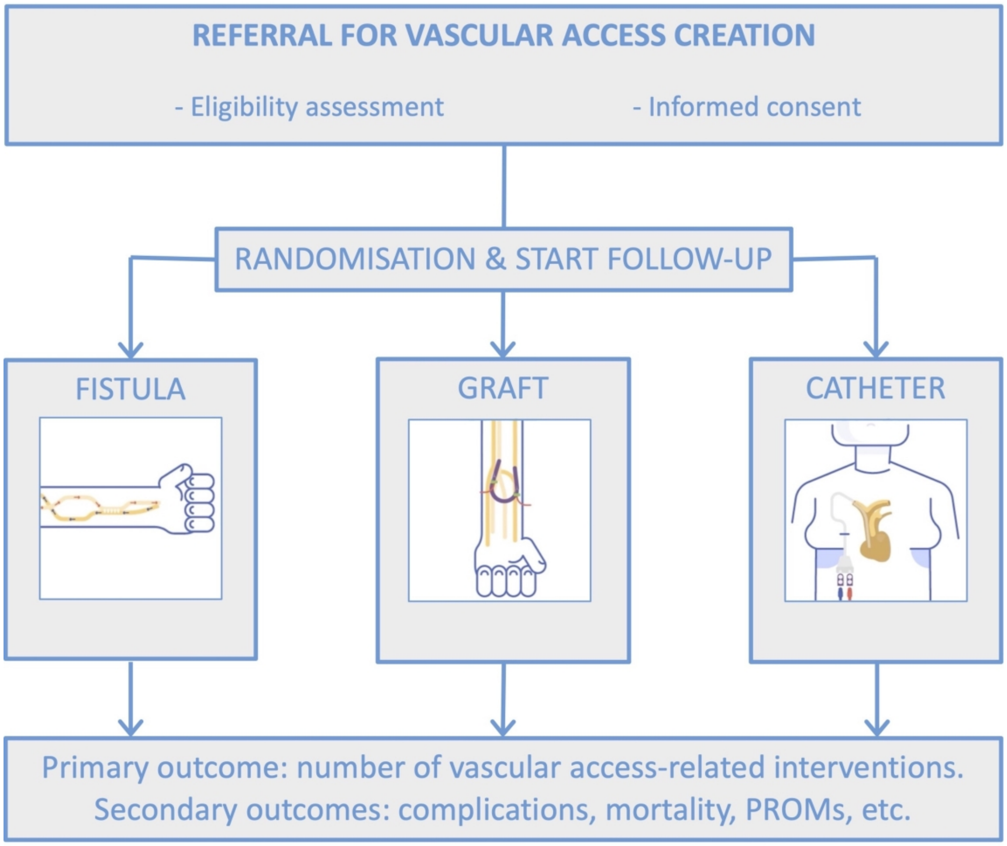

Study flow chart. On referral for vascular access creation, patients are screened for eligibility. Patients who meet the inclusion and exclusion criteria and provide informed consent will be randomised and allocated to one of the treatment strategies. Follow-up for the primary and secondary outcomes starts at the moment of treatment allocation. PROM: Patient-reported outcome measure.

279x237mm (300 x 300 DPI)

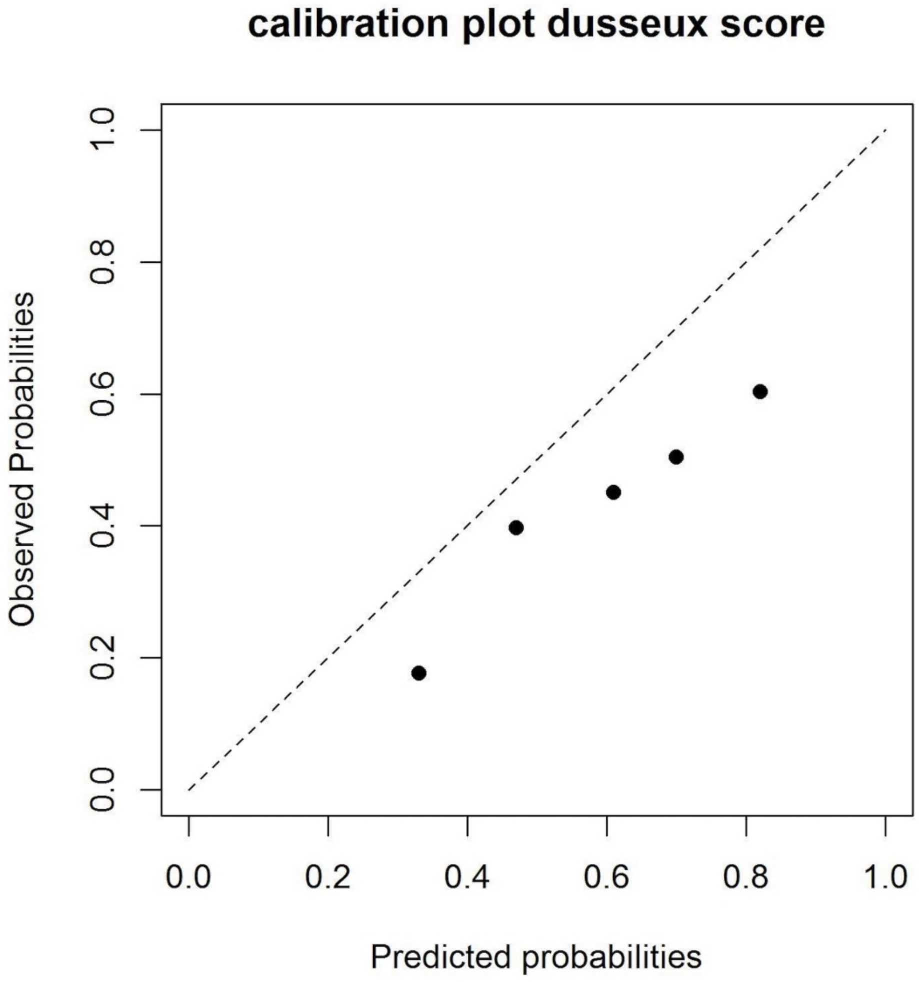

Calibration plot presenting the mortality risk as predicted by the Dusseux risk score and the observed risk in the NECOSAD cohort, such that the 45˚ line indicates perfect agreement between predicted and observed risks.

199x215mm (300 x 300 DPI)

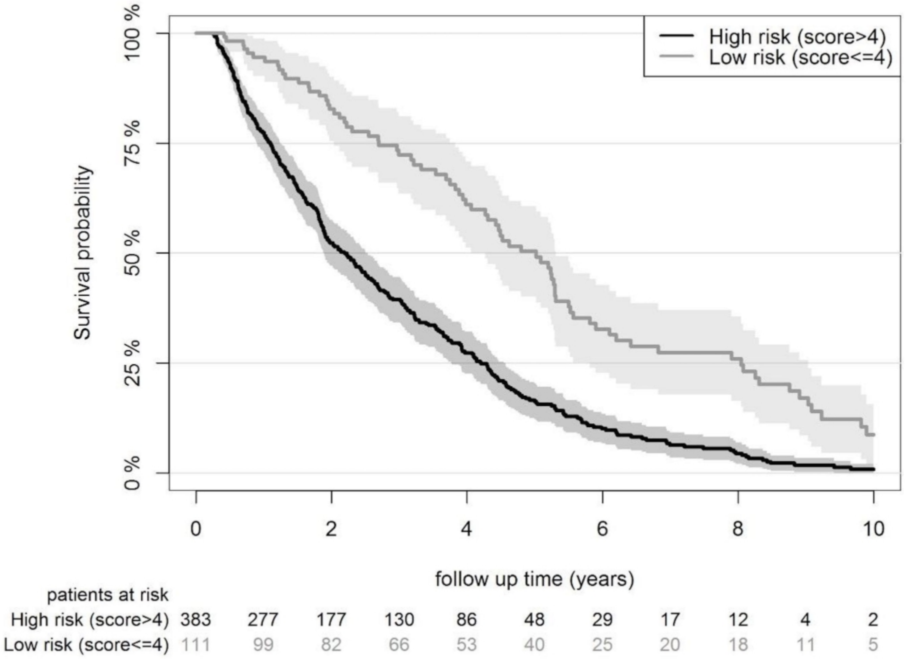

Kaplan Meier curve comparing the survival probability for the high risk and the low risk group in the NECOSAD cohort.

250x203mm (300 x 300 DPI)

Supplementary data 1: The Dusseux score with predictor definitions. [17]

Score item	Score points	Definition given in Dusseux article
Gender		
Male	1	
Female	0	
Age		
70-75	0	
75-80	2	
80-85	5	
≥85	9	
Diabetes		
Yes	2	
No	0	
Ischemic heart disease		history of coronary vascular disease, myocardial infarction, coronary artery bypass surgery, angioplasty, or abnormal angiography
Yes	2	
No	0	
PVD stage III or IV		Leriche-Fontaine classification
No	0	
Stage I or II	0	
Stage III or IV	5	
Cerebrovascular disease		
Yes	1	
No	0	
Chronic heart failure		New York Heart Association stages I–IV
No	0	
Stage I or II	2	
Stage III or IV	4	
Dysrhythmia		defined as atrial fibrillation and in the case of specific treatment with one or more antiarrhythmics, or the presence of a pacemaker or a cardiac implantable defibrillator
Yes	2	
No	0	
Chronic Respiratory disease		Including chronic respiratory failure, defined as a steady-state PaO2 o60mmHg regardless of the level of capnia, a sleep apnea syndrome or requiring oxygen therapy or home ventilatory assistance, and chronic obstructive pulmonary disease, defined as a cough with permanent or recurrent sputum, especially in the morning, 3 months/years for 2 consecutive years
Yes	2	
No	0	
Active malignancy		
Yes	5	
No	0	
Severe behavioural disorder		including dementia, psychosis, or severe neurosis that may affect patient dependence or compliance with treatment
Yes	6	
No	0	
Mobility		
Walks without help	0	
Needs assistance for transfers	4	
Totally dependent for transfers	9	
BMI		
<21	3	
21-25	1	
≥25	0	
CVC at dialysis initiation		
Yes	3	
No	0	

For peer review only

Reporting checklist for protocol of a clinical trial.

Based on the SPIRIT guidelines.

Instructions to authors

Complete this checklist by entering the page numbers from your manuscript where readers will find each of the items listed below.

Your article may not currently address all the items on the checklist. Please modify your text to include the missing information. If you are certain that an item does not apply, please write "n/a" and provide a short explanation.

Upload your completed checklist as an extra file when you submit to a journal.

In your methods section, say that you used the SPIRITreporting guidelines, and cite them as:

Chan A-W, Tetzlaff JM, Gøtzsche PC, Altman DG, Mann H, Berlin J, Dickersin K, Hróbjartsson A, Schulz KF, Parulekar WR, Krleža-Jerić K, Laupacis A, Moher D. SPIRIT 2013 Explanation and Elaboration: Guidance for protocols of clinical trials. BMJ. 2013;346:e7586

		Reporting Item	Page Number
**Administrative information**			
Title	#1	Descriptive title identifying the study design, population, interventions, and, if applicable, trial acronym	1

For peer review only

Trial registration	#2a	Trial identifier and registry name. If not yet registered, name of intended registry	2
Trial registration: data set	#2b	All items from the World Health Organization Trial Registration Data Set	2
Protocol version	#3	Date and version identifier	2
Funding	#4	Sources and types of financial, material, and other support	10
Roles and responsibilities: contributorship	#5a	Names, affiliations, and roles of protocol contributors	10
Roles and responsibilities: sponsor contact information	#5b	Name and contact information for the trial sponsor	1
Roles and responsibilities: sponsor and funder	#5c	Role of study sponsor and funders, if any, in study design; collection, management, analysis, and interpretation of data; writing of the report; and the decision to submit the report for publication, including whether they will have ultimate authority over any of these activities	10
Roles and responsibilities: committees	#5d	Composition, roles, and responsibilities of the coordinating centre, steering committee, endpoint adjudication committee, data management team, and	8

For peer review only

		other individuals or groups overseeing the trial, if applicable (see Item 21a for data monitoring committee)	
**Introduction**			
Background and rationale	#6a	Description of research question and justification for undertaking the trial, including summary of relevant studies (published and unpublished) examining benefits and harms for each intervention	3,4
Background and rationale: choice of comparators	#6b	Explanation for choice of comparators	3,4
Objectives	#7	Specific objectives or hypotheses	4
Trial design	#8	Description of trial design including type of trial (eg, parallel group, crossover, factorial, single group), allocation ratio, and framework (eg, superiority, equivalence, non-inferiority, exploratory)	4
**Methods: Participants, interventions, and outcomes**			
Study setting	#9	Description of study settings (eg, community clinic, academic hospital) and list of countries where data will be collected. Reference to where list of study sites can be obtained	4

For peer review only

Eligibility criteria	#10	Inclusion and exclusion criteria for participants. If applicable, eligibility criteria for study centres and individuals who will perform the interventions (eg, surgeons, psychotherapists)	4,5
Interventions: description	#11a	Interventions for each group with sufficient detail to allow replication, including how and when they will be administered	6,7
Interventions: modifications	#11b	Criteria for discontinuing or modifying allocated interventions for a given trial participant (eg, drug dose change in response to harms, participant request, or improving / worsening disease)	7
Interventions: adherance	#11c	Strategies to improve adherence to intervention protocols, and any procedures for monitoring adherence (eg, drug tablet return; laboratory tests)	7
Interventions: concomitant care	#11d	Relevant concomitant care and interventions that are permitted or prohibited during the trial	N/A
Outcomes	#12	Primary, secondary, and other outcomes, including the specific measurement variable (eg, systolic blood pressure), analysis metric (eg, change from baseline, final value, time to event), method of aggregation (eg, median, proportion), and time point for each outcome. Explanation of the clinical relevance of chosen efficacy and harm outcomes is strongly recommended	7,8

For peer review only

Participant timeline	#13	Time schedule of enrolment, interventions (including any run-ins and washouts), assessments, and visits for participants. A schematic diagram is highly recommended (see Figure)	4,5,9
Sample size	#14	Estimated number of participants needed to achieve study objectives and how it was determined, including clinical and statistical assumptions supporting any sample size calculations	8
Recruitment	#15	Strategies for achieving adequate participant enrolment to reach target sample size	4
**Methods: Assignment of interventions (for controlled trials)**			
Allocation: sequence generation	#16a	Method of generating the allocation sequence (eg, computer-generated random numbers), and list of any factors for stratification. To reduce predictability of a random sequence, details of any planned restriction (eg, blocking) should be provided in a separate document that is unavailable to those who enrol participants or assign interventions	5
Allocation concealment mechanism	#16b	Mechanism of implementing the allocation sequence (eg, central telephone; sequentially numbered, opaque,	5

For peer review only

		sealed envelopes), describing any steps to conceal the sequence until interventions are assigned	
Allocation: implementation	#16c	Who will generate the allocation sequence, who will enrol participants, and who will assign participants to interventions	5
Blinding (masking)	#17a	Who will be blinded after assignment to interventions (eg, trial participants, care providers, outcome assessors, data analysts), and how	4
Blinding (masking): emergency unblinding	#17b	If blinded, circumstances under which unblinding is permissible, and procedure for revealing a participant's allocated intervention during the trial	4
**Methods: Data collection, management, and analysis**			
Data collection plan	#18a	Plans for assessment and collection of outcome, baseline, and other trial data, including any related processes to promote data quality (eg, duplicate measurements, training of assessors) and a description of study instruments (eg, questionnaires, laboratory tests) along with their reliability and validity, if known. Reference to where data collection forms can be found, if not in the protocol	4,8

For peer review only

Data collection plan: retention	#18b	Plans to promote participant retention and complete follow-up, including list of any outcome data to be collected for participants who discontinue or deviate from intervention protocols	9
Data management	#19	Plans for data entry, coding, security, and storage, including any related processes to promote data quality (eg, double data entry; range checks for data values). Reference to where details of data management procedures can be found, if not in the protocol	8
Statistics: outcomes	#20a	Statistical methods for analysing primary and secondary outcomes. Reference to where other details of the statistical analysis plan can be found, if not in the protocol	8,9
Statistics: additional analyses	#20b	Methods for any additional analyses (eg, subgroup and adjusted analyses)	8,9
Statistics: analysis population and missing data	#20c	Definition of analysis population relating to protocol non-adherence (eg, as randomised analysis), and any statistical methods to handle missing data (eg, multiple imputation)	9

Methods: Monitoring

Data monitoring: formal committee	#21a	Composition of data monitoring committee (DMC); summary of its role and reporting structure; statement of whether it is independent from the sponsor and competing interests; and reference to where further	8

For peer review only

		details about its charter can be found, if not in the protocol. Alternatively, an explanation of why a DMC is not needed	
Data monitoring: interim analysis	#21b	Description of any interim analyses and stopping guidelines, including who will have access to these interim results and make the final decision to terminate the trial	8
Harms	#22	Plans for collecting, assessing, reporting, and managing solicited and spontaneously reported adverse events and other unintended effects of trial interventions or trial conduct	8
Auditing	#23	Frequency and procedures for auditing trial conduct, if any, and whether the process will be independent from investigators and the sponsor	N/A
**Ethics and dissemination**			
Research ethics approval	#24	Plans for seeking research ethics committee / institutional review board (REC / IRB) approval	9
Protocol amendments	#25	Plans for communicating important protocol modifications (eg, changes to eligibility criteria, outcomes, analyses) to relevant parties (eg, investigators, REC / IRBs, trial participants, trial registries, journals, regulators)	N/A

For peer review only

Consent or assent	#26a	Who will obtain informed consent or assent from potential trial participants or authorised surrogates, and how (see Item 32)	5
Consent or assent: ancillary studies	#26b	Additional consent provisions for collection and use of participant data and biological specimens in ancillary studies, if applicable	N/A
Confidentiality	#27	How personal information about potential and enrolled participants will be collected, shared, and maintained in order to protect confidentiality before, during, and after the trial	8
Declaration of interests	#28	Financial and other competing interests for principal investigators for the overall trial and each study site	10
Data access	#29	Statement of who will have access to the final trial dataset, and disclosure of contractual agreements that limit such access for investigators	10
Ancillary and post trial care	#30	Provisions, if any, for ancillary and post-trial care, and for compensation to those who suffer harm from trial participation	N/A
Dissemination policy: trial results	#31a	Plans for investigators and sponsor to communicate trial results to participants, healthcare professionals, the public, and other relevant groups (eg, via publication, reporting in results databases, or other data sharing arrangements), including any publication restrictions	10

For peer review only

Dissemination policy: authorship	#31b	Authorship eligibility guidelines and any intended use of professional writers	10
Dissemination policy: reproducible research	#31c	Plans, if any, for granting public access to the full protocol, participant-level dataset, and statistical code	10

Appendices

Informed consent materials	#32	Model consent form and other related documentation given to participants and authorised surrogates	N/A
Biological specimens	#33	Plans for collection, laboratory evaluation, and storage of biological specimens for genetic or molecular analysis in the current trial and for future use in ancillary studies, if applicable	N/A

None The SPIRIT Explanation and Elaboration paper is distributed under the terms of the Creative Commons Attribution License CC-BY-NC. This checklist can be completed online using https://www.goodreports.org/, a tool made by the EQUATOR Network in collaboration with Penelope.ai

For peer review only
